# Sustainable gold nanoparticles using *Jania rubens*, targeted therapeutic potential in prostate cancer and mechanistic insights via GO/KEGG pathway analysis

**DOI:** 10.1038/s41598-026-56865-z

**Published:** 2026-07-11

**Authors:** Noura El-Ahmady El-Naggar, Eman M. Sarhan, Banan Saher Amien, Munera Wael Ibrahim, Yasmine Yousry Elsaeed, Asmaa A. El-Sawah

**Affiliations:** 1 Department of Bioprocess Development, Genetic Engineering and Biotechnology Research Institute, City of Scientific Research and Technological Applications (SRTA-City), New Borg El-Arab, Alexandria 21934 Egypt; 2 Biochemistry Department, Faculty of Science, Alexandria University, Alexandria, Egypt; 3 Biotechnology and Its Application Program, Department of Botany, Faculty of Science, Mansoura University, Mansoura, 35516 Egypt; 4 Clinical Pathology Department, Official Mansoura University Hospital, Mansoura, Egypt

**Keywords:** AuNPs, *Jania rubens*, GC analysis, Characterization, Optimization, Anti-prostate cancer, GO/KEGG pathway, Ehrlich ascites carcinoma (EAC), Biochemistry, Biological techniques, Biotechnology, Cancer, Computational biology and bioinformatics, Drug discovery

## Abstract

Gold nanoparticles (AuNPs) are highly versatile nanomaterials due to their exceptional physicochemical and biological properties with promising biomedical applications. This study presents a rapid, eco-friendly biosynthesis of AuNPs using the red alga *Jania rubens* (Jan-AuNPs). GC analysis of the algal extract revealed a total fatty acid content of 1.41 mg/g DW, dominated by saturated fatty acids primarily methyl palmitate (53.22%) and methyl stearate (27.89%) alongside methyl oleate (9.42%). Phytochemical profiling demonstrated a rich biochemical composition, including a high total phenolic content of 299.82 ± 1.83 mg GAE/g DW, total flavonoid content of 35.61 ± 0.52 mg QE/g DW, and total carbohydrate content (TCC) of 166 ± 1.05 mg GE/g DW, collectively conferring potent reducing, capping and stabilizing capacities of the algal extract in mediating Jan-AuNPs biosynthesis. Formation of Jan-AuNPs was visually confirmed by a color change from pale yellow to ruby red or pinkish-red, which was further confirmed by UV–vis spectroscopy with a surface plasmon resonance (SPR) peak at 549 nm. TEM analysis revealed predominantly spherical particles with an average diameter of 16.26 nm, with a few rod-shaped particles also detected. The crystalline nature of Jan-AuNPs was validated by XRD and SAED analyses. Zeta potential measurements revealed a surface charge of -28 mV, indicating good colloidal stability. FTIR analysis confirmed the active involvement of diverse algal biomolecules in the formation and stabilization of Jan-AuNPs. Biosynthesis conditions were optimized using face-centered central composite design (FCCCD), achieving the highest yield of 289 µg/mL at pH 7, 60 °C, 3 h incubation, and 300 µg/mL gold ion concentration. *In silico* predictive analysis identified cancer-associated gene targets modulated by AuNPs, thereby predicting prostate cancer (PC) as the malignancy with the highest therapeutic susceptibility for AuNPs-based interventions. Experimental validation confirmed potent and selective antitumor activity against PC3 prostate cancer cells *in vitro* (IC_50_ = 6.39 µg/mL; SI = 15.24) with minimal cytotoxicity toward normal HFB4 cells (IC_50_ = 97.41 µg/mL). *In vivo* evaluation using the Ehrlich ascites carcinoma (EAC) model in Swiss albino mice demonstrated that combined treatment with Jan-AuNPs and doxorubicin achieved a tumor growth inhibition of 97.33%. These findings establish *Jania rubens*-mediated biosynthesis as a sustainable and scalable platform for producing bioactive AuNPs and highlight the pivotal role of bioinformatics in guiding experimental cancer nanomedicine research. The results highlight Jan-AuNPs as a promising, safe, and multi-targeted nanotherapeutic candidate with significant potential for the management of prostate cancer.

## Introduction

Cancer is a life-threatening disease marked by uncontrolled cell division that can invade nearby tissues and spread via the lymphatic or systemic circulation. According to the WHO, cancer caused 9.7 million deaths and 20 million new cases in 2022. By 2050, new cases are expected to exceed 35 million, a 77% increase. Prostate cancer (PC) alone accounted for 1.41 million new cases and 375,000 deaths globally in 2020, making it the second most common cancer and the fifth leading cause of cancer death in men^1^, following lung cancer. Current therapeutic strategies used to treat PC include hormone therapy, chemotherapy, targeted therapy, and targeted protein degradation therapy. Although these therapies can be effective during early treatment, the development of drug resistance remains a major challenge. Hormone therapy can temporarily delay disease progression; however, mutations, variant expressions within the androgen receptor signaling pathway, and gene amplification ultimately reduce its long-term effectiveness. Chemotherapy is limited by its severe toxic side effects and the rapid emergence of resistance. More recent strategies, including targeted therapy and targeted protein degradation therapy, offer promising potential; however, their clinical impact is restricted by pharmacokinetic challenges, redundancy of signaling pathways, and tumor heterogeneity, which still pose significant barriers to successful clinical use^2^. Therefore, there is an urgent need for novel therapeutic strategies that combine high selectivity with minimal side effects.

Nanotechnology offers a promising avenue to overcome these limitations by improving targeted drug delivery, enhancing therapeutic efficacy, and reducing systemic toxicity. Nanoparticles (NPs) are typically classified as either organic or inorganic. Organic NPs include various structures such as micelles, dendrimers, liposomes, nanogels, polymeric nanoparticles, and layered biopolymers. Among inorganic nanoparticles , metallic nanoparticles are the most widely studied, including gold (Au), cerium (Ce), silver (Ag), platinum (Pt), palladium (Pd), copper (Cu), nickel (Ni), selenium (Se), iron (Fe), gadolinium (Gd), and their respective oxides^3^. Gold nanoparticles (AuNPs) have gained significant attention as one of the most extensively studied and applied nanomaterials in modern science and technology, primarily due to their distinctive physical, chemical, and biological properties, excellent biocompatibility, and low toxicity at controlled doses^4^. Their high surface-area-to-volume ratio facilitates the conjugation of various biomolecules, such as drugs, nucleic acids, proteins, and antibodies, thereby enabling targeted drug delivery. Additionally, AuNPs can be synthesized in diverse shapes (including spheres, rods, cubes, and stars) and sizes, both of which significantly influence their optical, catalytic, and biological behaviors. One of the most notable features of AuNPs is their optical property known as surface plasmon resonance (SPR). This phenomenon results in strong absorption and scattering in the visible to near-infrared spectrum, enabling their use in imaging, sensing, and photothermal applications.

Owing to these unique attributes, AuNPs offer a versatile and promising platform for cancer therapy^5^, particularly in the treatment of prostate cancer. They serve as effective carriers for anticancer drugs and can be employed in photothermal therapy, where localized heating induced by light absorption destroys cancerous cells. Furthermore, the ability of AuNPs to selectively bind to biological markers enhances their applicability in biosensors and optical bioimaging techniques. AuNPs are widely utilized in immunoassays, clinical diagnostics, and gene delivery. They have also shown potential in the treatment of rheumatoid arthritis, viral infections, and microbial infections^6^. Moreover, AuNPs show great promise in managing diabetes and its related microvascular complications, owing to their anti-hyperglycemic activity. They also exhibit antioxidant, anti-inflammatory, wound healing, anti-fibrotic, and antiangiogenic properties^7^.

Traditionally, AuNPs have been synthesized using physical and chemical methods. Chemical reduction is the most widely used method, where gold salts (HAuCl_4_) are reduced using agents such as sodium citrate or sodium borohydride in the presence of stabilizers. Physical methods such as laser ablation, thermal decomposition, and photochemical methods are also employed. However, these approaches pose several limitations. Chemical synthesis often involves hazardous or carcinogenic substances, restricting their suitability for therapeutic use^8^. Physical methods, on the other hand, require high energy input and expensive equipment. Furthermore, both methods commonly face challenges such as poor control over crystal growth, aggregation, and low stability of the produced nanoparticles^9^. To overcome these drawbacks, green synthesis has emerged as an eco-friendly and sustainable approach to produce metal nanoparticles using biological resources. AuNPs can be biosynthesized using various natural biological sources such as plant extracts and microorganisms, including bacteria, fungi, actinomycetes, and algae, which act as effective nanofactories^10^. This green synthesis approach offers several advantages, as it employs non-toxic reducing agents, is cost-effective and energy-efficient, allows for controlled nanoparticles’ size, and utilizes renewable resources^11,12^. These natural materials serve as both reducing and stabilizing agents, aligning with the principles of green chemistry and minimizing environmental impact. Among these sources, marine algae have recently attracted considerable attention as efficient biological nanofactories for the green synthesis of nanoparticles due to their simplicity, safety, and cost-effectiveness. Their richness in bioactive compounds, including proteins, polysaccharides, polyphenols, and pigments, enables a simple, biocompatible, and sustainable synthesis process, as these constituents act simultaneously as reducing and capping agents, making it particularly suitable for biomedical and pharmaceutical applications^13^. Among them, red algae (Rhodophyta) have been particularly recognized for their ability to mediate the synthesis of various metallic nanoparticles exhibiting enhanced biological activity and stability. Many algal species are efficient in the synthesis of metal/metal oxide NPs^14^. In addition to *L. flaviatilis* and *C. officinalis*, numerous additional red algae species, such as *Kappaphycus alvarezii*, *Galaxaura elongata*, and *Chondrus crispus*, have been documented to efficiently biosynthesize AuNPs^15^.

This study aims to biosynthesize Jan-AuNPs using the hot water extract of *Jania rubens* (*J. rubens*), optimize the biosynthesis process through a Face-Centered Central Composite Design (FCCCD), and characterize the synthesized nanoparticles using UV–Vis spectroscopy, TEM, EDX, XRD, FTIR, and zeta potential analyses. Additionally, the study investigates the *in vivo*, *in vitro*, and *in silico* cytotoxicity and antitumor activity of the Jan-AuNPs. To the best of our knowledge, this is the first report on the biosynthesis of Jan-AuNPs using the hot water extract of *J. rubens*, a red marine alga known for its high content of bioactive compounds, including carrageenan, phenolics, and flavonoids, which exhibit antioxidant, antimicrobial, and anticancer properties.

## Materials and methods

**Fig. 1:**
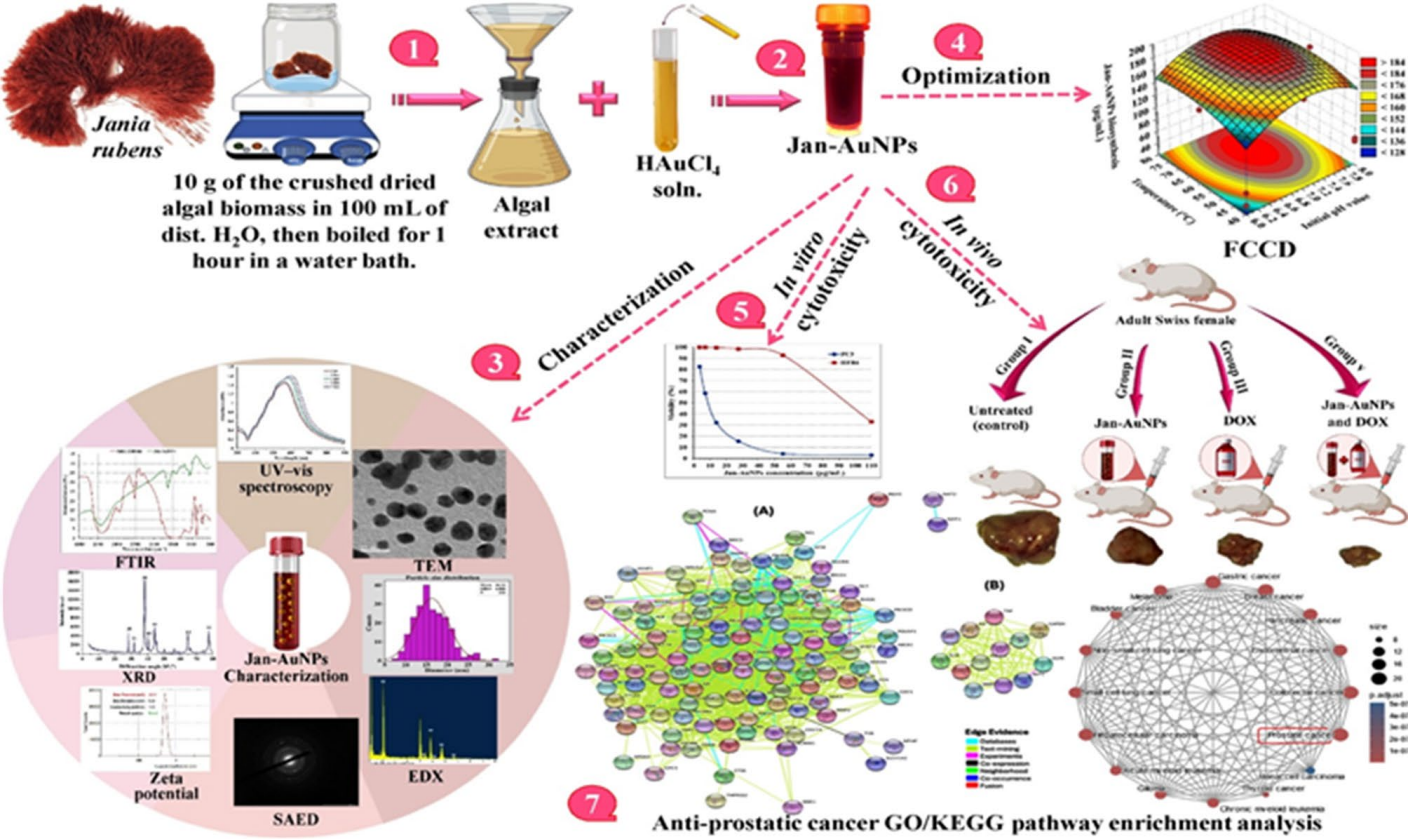
A schematic overview of the research design framework employed in this study.

Figure 1 shows a schematic illustration of the research design framework used in this investigation.

### Collection and preparation of the extract of *J. rubens*

The biomass of *J. rubens* was collected in June 2024 from the beaches of Hurghada, Red Sea Governorate, Egypt (27°15’0” N and 33°49’0” E) during low tide at inter-tidal zone from shoreline. The collected *J. rubens* biomass underwent a thorough cleaning process, initially rinsed with seawater followed by fresh water to eliminate any sand, salt deposits, and other contaminants. The cleaned algae were then air-dried at room temperature. For extract preparation, approximately 10 g of the dried algal biomass was mixed with 100 mL of distilled water in a closed system and heated to 80 °C for 1 h. The resulting mixture was subsequently filtered using Whatman Qualitative Filter Paper, Grade 1 then centrifuged at 4000*×g*, and the supernatant (approximately 90 mL) was collected and stored at 4 °C for use in further experiments.

### The phytochemical characterization of the extract of *J. rubens*

#### Fatty acids extraction and determination

Fatty acid profiling was performed following the one-step direct methylation protocol described by Liu et al.^16^ using acid-catalyzed transesterification. To extract fatty acids from the sample, approximately 100 mg of the sample is weighed into a PTFE-lined test tube, followed by the addition of 2% methanolic H₂SO₄. The mixture is then heated at 60 °C for 5 h and allowed to cool. Once cooled, 1 mL of distilled water and 1 mL of hexane are added to the tube. The sample is vortexed for one minute to ensure thorough mixing, after which the upper hexane layer is carefully transferred to a GC vial for subsequent determination. The analysis is performed using a Scion (formerly Varian) 456-GC gas chromatograph equipped with an 8400 autosampler, a split/splitless injector, and a flame ionization detector (FID). The separation column used is an RT-2560 capillary column with a length of 100 m, an internal diameter of 0.25 mm, and a film thickness of 0.20 μm. The injector and detector temperatures are set at 240 °C and 255 °C, respectively. Helium is used as the carrier gas under constant flow mode at a column flow rate of 0.80 mL/min. The oven is programmed with a multi-ramp temperature gradient: it begins at an initial temperature of 50 °C held for 4 min, then rises at a rate of 8 °C/min to 180 °C and is held for 25 min. It is subsequently increased at 1 °C/min to 185 °C, held for 15 min, and finally ramped at 25 °C/min to 220 °C where it is held for 31 min. Quantification of fatty acids was achieved using a standard mixture containing 37 FAMEs^16^.

#### Determination of carbohydrates content (TCC)

The TCC of the *J. rubens* extract was quantified using the anthrone colorimetric assay. In brief, 200 µL of each sample was reacted with 5 mL of freshly prepared anthrone reagent (2 g anthrone dissolved in 1 L of concentrated sulfuric acid; Techno Pharmchem, India). The reaction mixture was incubated at 90 °C for 17 min, allowed to cool to room temperature, and the absorbance was recorded at 620 nm against a reagent blank. Carbohydrate concentration was calculated from a glucose calibration curve generated from a 200 µg/mL stock solution (El-Nasr Pharmaceutical Chemicals, Egypt). Data were expressed as mg glucose equivalent per gram of sample (mg GE/g). All determinations were carried out in triplicate.

#### Determination of total phenolic content (TPC)

The TPC of the *J. rubens* extract was quantified using the Folin–Ciocalteu colorimetric assay with minor modifications. Briefly, 0.5 mL of suitably diluted extract was mixed with 2.5 mL of 10% (v/v) Folin–Ciocalteu reagent (Fluka, Biochemical Inc., Bucharest, Romania). After allowing the mixture to react for 5 min at room temperature, 2.0 mL of 7.5% (w/v) sodium carbonate solution was added. The reaction mixture was vortexed thoroughly and incubated in the dark at room temperature for 30 min to ensure complete color development. Absorbance was subsequently recorded at 765 nm using a UV–Vis spectrophotometer against a reagent blank. A standard calibration curve was prepared using 0–200 µg/mL of gallic acid (Biomedical Inc., Orange City, FL, USA), and results were expressed as milligrams of gallic acid equivalents per gram of dry weight (mg GAE/g DW). All analyses were conducted in triplicate and presented as mean ± standard deviation.

#### Determination of total flavonoid content (TFC)

The TFC of the *J. rubens* extract was estimated using the aluminum chloride colorimetric assay. In brief, 0.5 mL of the extract was combined with 1.5 mL of methanol, followed by the addition of 0.1 mL of 10% (w/v) aluminum chloride, 0.1 mL of 1 M potassium acetate, and 2.8 mL of distilled water. The reaction mixture was allowed to stand at room temperature for 30 min to ensure complete complex formation. Absorbance was then measured at 415 nm against a reagent blank prepared without aluminum chloride. Quercetin (0–100 µg/mL; El-Nasr Pharmaceutical Chemicals, Cairo, Egypt) served as the standard for constructing the calibration curve. The total flavonoid content was calculated from the standard curve and expressed as milligrams of quercetin equivalents per gram of dry weight (mg QE/g DW). All determinations were performed in triplicate, and results were reported as mean ± standard deviation.

#### Biosynthesis of Jan-AuNPs

Gold chloride trihydrate (HAuCl_4_·3 H₂O; Sigma-Aldrich) was prepared by dissolving the salt in distilled water to obtain a final concentration of 1000 µg/mL. For nanoparticle synthesis, 3 mL of the *J. rubens* aqueous extract was mixed with 1 mL of the gold chloride solution. The reaction mixture was incubated in the dark at 60 °C for 3 h. A visible color change from pale yellow to dark red indicated the reduction of Au³⁺ ions and the successful formation of Jan-AuNPs.The resulting Jan-AuNPs were centrifuged at 11,000*×g* for 15 min. The pellets of Jan-AuNPs were washed thoroughly with distilled water, and the supernatant containing residual algal extract was discarded. This washing step was repeated three times to ensure complete removal of unreacted biomolecules. The purified Jan-AuNPs were either lyophilized or re-dispersed in distilled water for subsequent characterization and applications.

#### Characterization of the biosynthesized Jan-AuNPs

The biosynthesized Jan-AuNPs were monitored at various time intervals using a UV–Visible spectrophotometer (TI Unicam 5625 UV/VIS, Vision Software V3.20, UK) to detect the surface plasmon resonance (SPR) and confirm nanoparticles’ formation, with maximum absorbance typically observed between 500 and 600 nm.

The morphology, size, and structural features of Jan-AuNPs were analyzed using Transmission Electron Microscopy (TEM; JEOL JEM-2100 Plus, Ltd., Japan). The crystalline nature of the nanoparticles was confirmed via Selected Area Electron Diffraction (SAED) patterns, also obtained using the TEM apparatus. Elemental composition and the presence of constituent elements in the Jan-AuNPs were identified through Energy Dispersive X-ray spectroscopy (EDX), while elemental mapping was performed using TEM to visualize the distribution and composition of the biosynthesized Jan-AuNPs.

The hydrodynamic diameter and size distribution of the biosynthesized Jan-AuNPs were determined by Dynamic Light Scattering (DLS), and the surface charge (zeta potential) was measured using a Malvern Zetasizer Nano ZS (Malvern Panalytical, UK), providing insights into the colloidal stability of the Jan-AuNPs in aqueous solutions.

Fourier Transform Infrared Spectroscopy (FTIR) was employed to identify the functional groups involved in the reduction and stabilization of Jan-AuNPs by analyzing functional group alterations. FTIR spectra were recorded in the range of 4500–500 cm⁻¹ at a resolution of 1 cm^− 1^.

Crystallographic analysis of the Jan-AuNPs was carried out using X-ray Diffraction (XRD) with a Bruker D2 Phaser (2nd Generation) diffractometer equipped with CuKα radiation (λ = 1.5406 Å), operated at 10 kV and 30 mA. XRD patterns were collected over a 2*θ* range of 10°–70° at a scanning rate of 2°/min, providing information on the crystalline nature and structural properties of the synthesized Jan-AuNPs.

#### Face-centered central composite design optimization

A Face-Centered Central Composite Design (FCCCD) was used to maximize the biosynthesis of Jan-AuNPs by evaluating both individual and interaction effects of four key bioprocess variables: initial pH (A), temperature (B), incubation time (C), and gold ion concentration (D). A total of 30 experimental runs were conducted to explore the effects of various combinations of pH, temperature, incubation time, and gold concentration. Each independent variable was tested at three levels: low (− 1), central (0), and high (+ 1). The central (zero) levels chosen for the experimental design were as follows: gold ion concentration of 200 µg/mL, temperature of 60 °C, initial pH of 7, and incubation time of 3 h. The second-order polynomial equation is used to determine the influence and interaction of these independent variables and the yield of Jan-AuNPs as follows:

1 $$Y = {\beta _0} + \sum\limits_i {{\beta _i}{X_i}} + \sum\limits_{ii} {{\beta _{ii}}{X_i}^2} + \sum\limits_{ij} {{\beta _{ij}}{X_i}{X_j}}$$

where Y is the predicted Jan-AuNPs yield, β_0_ represents the regression coefficients, β_i_ represents the linear coefficient, β_ii_ represents the quadratic coefficients, β_ij_ represents the interaction coefficients). X_i_ and Xⱼ are the coded levels of the independent variables (A, B, C, D).

#### *In vitro* anticancer activity evaluation of the biosynthesized Jan-AuNPs

The *in vitro* cytotoxic and anticancer potential of the biosynthesized Jan-AuNPs was evaluated using the colorimetric MTT assay, based on the method described by Mosmann^17^. The assay was performed against the human prostate cancer cell line PC3 (ATCC: CRL-1435), representing grade IV prostatic adenocarcinoma, and the human normal skin fibroblast cell line HFB4 (BBRJ code: nh-skp-fb0040) as a non-cancerous control. Cells were seeded in a 96-well tissue culture plate at a density of 1 × 10⁵ cells/mL (100 µL/well) and incubated at 37 °C in a humidified atmosphere containing 5% CO₂ for 24 h to allow the formation of a complete monolayer. After incubation, the culture medium was removed, and the wells were gently washed twice with wash media to eliminate non-adherent cells. Serial two-fold dilutions of Jan-AuNPs were prepared in RPMI medium supplemented with 2% serum (maintenance medium). Prior to application, the Jan-AuNPs were filtered through a 0.45 μm syringe filter. Cells were treated with varying concentrations of 0.1 mL Jan-AuNPs (110, 55, 27.5, 13.75, 6.875, and 3.4375 µg/mL). Three wells per plate were used as untreated controls and received only maintenance medium. The plates were incubated at 37 °C with 5% CO₂ and 100% relative humidity for 24 h. Following treatment, cells were observed under an inverted microscope for morphological changes indicative of cytotoxicity, including partial or whole loss of the monolayer integrity, cell rounding, shrinkage, and cell granulation. Subsequently, 20 µL of MTT solution (5 mg/mL in PBS; Bio Basic Canada Inc.) was added to each well. Plates were placed on a shaking platform at 150 rpm for 5 min to ensure proper mixing, then incubated for 4 h at 37 °C with 5% CO_2_ to allow the formation of formazan crystals via mitochondrial reduction of MTT. After incubation, the media was removed carefully, and formazan crystals (MTT metabolic product) were solubilized in 200 µL of DMSO. Plates were again placed on a shaker at 150 rpm for 5 min to ensure a complete mix of the formazan into the solvent. The optical density was measured at 560 nm with background correction at 620 nm using a microplate reader. The following formula is applied to estimate the cell viability percentage:

2 $${\mathrm{Viability}}\% = \left( {{\mathrm{O}}{{\mathrm{D}}_{{\mathrm{test}}}}/{\mathrm{O}}{{\mathrm{D}}_{{\mathrm{control}}}}} \right) \times {\text{ 1}}00$$

3 $${\text{Cytotoxicity }}\% = {\text{ 1}}00{\text{ }}{-}{\text{ Viability }}\%$$

#### *In-vivo* cytotoxicity of the biosynthesized Jan-AuNPs using the Ehrlich solid tumor

Adult female Swiss albino mice (9–10 weeks old; weighing 25–30 g) were obtained from the Theodore Bilharz Research Institute, Giza, Egypt. The animals were housed in standard polycarbonate cages under controlled environmental conditions: temperature of 25–26 °C, relative humidity of 20%, and a 12-hour light/dark cycle. Standard diet and water were available ad libitum for the duration of the experiment. Ehrlich ascites carcinoma (EAC) cells, obtained from the American Type Culture Collection (ATCC) through the Holding Company for Biological Products and Vaccines (VACSERA), Cairo, Egypt, were used to induce tumors. Each mouse received a subcutaneous injection of 2 × 10^6^ EAC cells to initiate Ehrlich solid tumor (EST) formation. Mice were divided into four groups of eight mice each. Tumors developed within five days post-injection, having a long diameter average of 0.65 ± 0.02 cm and a short diameter average of 0.47 ± 0.02 cm by the start of treatment (day 0): Group I: Untreated tumor-bearing control. Group II: Treated with doxorubicin (DOX) at 2 mg/kg/day. Group III: Treated with Jan-AuNPs at 2 mg/kg/day. Group IV: Treated with a combination of DOX and Jan-AuNPs. All treatments were administered every five days for a total of 20 days. To manage pain associated with tumor development, meloxicam (5 mg/kg, subcutaneously; Adwic-El Nasr Pharmaceutical Co.) was administered daily starting from the day of tumor induction and continued as needed based on veterinary evaluation. All mice procedures, including euthanasia and anesthesia, were carried out in compliance with international ethical standards and the Institutional Animal Care and Use Committee’s (IACUC) guidelines. Strict adherence to humane endpoints was maintained to minimize animal suffering. These criteria included signs of severe pain or distress, such as > 20% body weight loss, extreme lethargy, prolonged appetite loss, or decreased mobility, which were used to determine when an animal should be withdrawn from the study or humanely euthanized. No visible signs of toxicity or > 20% weight loss was detected in any group during the 20-day treatment period. On day 21, the mice were anesthetized with thiopental sodium (40 mg/kg; Pharmadrug Production GmbH) and sacrificed via cervical dislocation. Tumor size was measured once after excision at the end of the experiment using a vernier caliper, then preserved in buffered formalin for histopathological analysis. Tumor volume (cm³) was calculated using the formula:

4 $${\mathrm{V}} = \left( {{\mathrm{L}} \times {{\mathrm{S}}^{\mathrm{2}}}} \right) \times {\text{ }}0.{\mathrm{5}}$$

Where *L* is the longest diameter (cm) and *S* is the shortest perpendicular diameter (cm). To represent the data, we expressed the results as mean ± standard deviation (SD) for each experimental group, which accounts for the biological variability among mice.

Tumor growth inhibition was calculated according to Schirner et al.^18^:

5 $$\:\mathrm{G}\mathrm{r}\mathrm{o}\mathrm{w}\mathrm{t}\mathrm{h}\:\mathrm{i}\mathrm{n}\mathrm{h}\mathrm{i}\mathrm{b}\mathrm{i}\mathrm{t}\mathrm{i}\mathrm{o}\mathrm{n}\:\left(\mathrm{\%}\right)\hspace{0.17em}=\hspace{0.17em}100\:\--\:(\:\frac{{\Delta\:}\mathrm{T}}{{\Delta\:}\mathrm{C}}\:\times\:100)$$

Where ΔT is the change in tumor volume in the treated group and ΔC in the control group. Histological sections were stained with hematoxylin and eosin and examined under a light microscope.

### Ethical approval

All experimental protocols were approved by the Animal Care and Use Committee of Mansoura University (MU-ACUC), Egypt (Approval Code: MU-ACUC SC.R.25.04.26). All procedures complied with national regulations and ARRIVE guidelines for animal research.

### *In silico* protein-protein interaction (PPI) and GO/KEGG enrichment analysis

#### *In silico* computational analysis

##### AuNPs-related target prediction

Nanogold predicted targets were collected using the STRING (https://string-db.org/, accessed on 6 Apr 2025) text-mining PubMed query search of ‘Nanogold’, Gold nanoparticle’, ‘AuNP’, or ‘Au-NP’ keywords, individually. Then, all retrieved data were combined after removing irrelevant and duplicated targets to construct a single ‘AuNPs’ dataset.

##### Cancer-related targets’ prediction

Cancer-related targets were retrieved from two human disease/gene databases, Disease (https://diseases.jensenlab.org/, accessed on 6 Apr 2025), and GeneCards (https://www.genecards.org/, accessed on 6 Apr 2025), using the keywords ‘Cancer’, ‘Carcinoma’, and Carcinogenesis’. The top highly scored 250 targets from each dataset query were selected individually and merged after removing duplicate targets to construct a single ‘Cancer’ dataset.

##### Protein–protein interaction (PPI) network construction and analysis

A Venn diagram was used to represent the common targets intersecting volume of both cancer and AuNPs datasets (https://bioinfogp.cnb.csic.es/tools/venny/, accessed on 9 Apr 2025). The PPI network of Cancer/ AuNPs’ common targets was constructed using the STRING v.12.0 database (https://string-db.org/, accessed on 12 Apr 2025). A list of 120 ENTREZID was used as input identifiers, and the analysis parameters were adjusted to set *Homo sapiens* organism species at a high confidence score > 0.7. The generated data was imported into Cytoscape v.3.10.3 software for further analysis.

##### GO/KEGG pathway enrichment analysis

Gene ontology (GO) and pathway functional significance of Cancer/ AuNPs’ common targets were explored by *clusterProfiler* v4.12.6 R Bioconductor package enrichment analysis (https://bioconductor.org/packages/release/bioc/html/clusterProfiler.html). SYMBOL gene identifiers (IDs) of the common targets were converted into ENTREZID using the human annotation database (*org.Hs.eg.db* v3.19.1) by *bitr* mapping function. GO terms for biological processes (BP), cellular components (CC), and molecular function (MF) were identified using the *groupGO* function at the hierarchy depth of level 3. Kyoto Encyclopedia of Genes and Genomes (KEGG) pathway enriched analysis was performed using *enrichKEGG* at FDR *P*.adjust < 0.05, *P*-value < 0.05, and q-value < 0.2. Cancer types were extracted using the KEGG pathway ID of ‘hsa052XX’. KEGG modules were explored using the *enrichMKEGG* function. Cancer types were extracted and sorted in ascending order by “p.adjust” and descending by FoldEnrichment to select the most significant cancer type pathway. ggplot2 v.3.5.1 R package was used to represent the enrichment plots. The *browseKEGG* function of the *clusterProfiler* R package was used to visualize the KEGG pathway gene expression map.

## Results and discussion

### GC of *J. rubens* extract

**Fig. 2:**
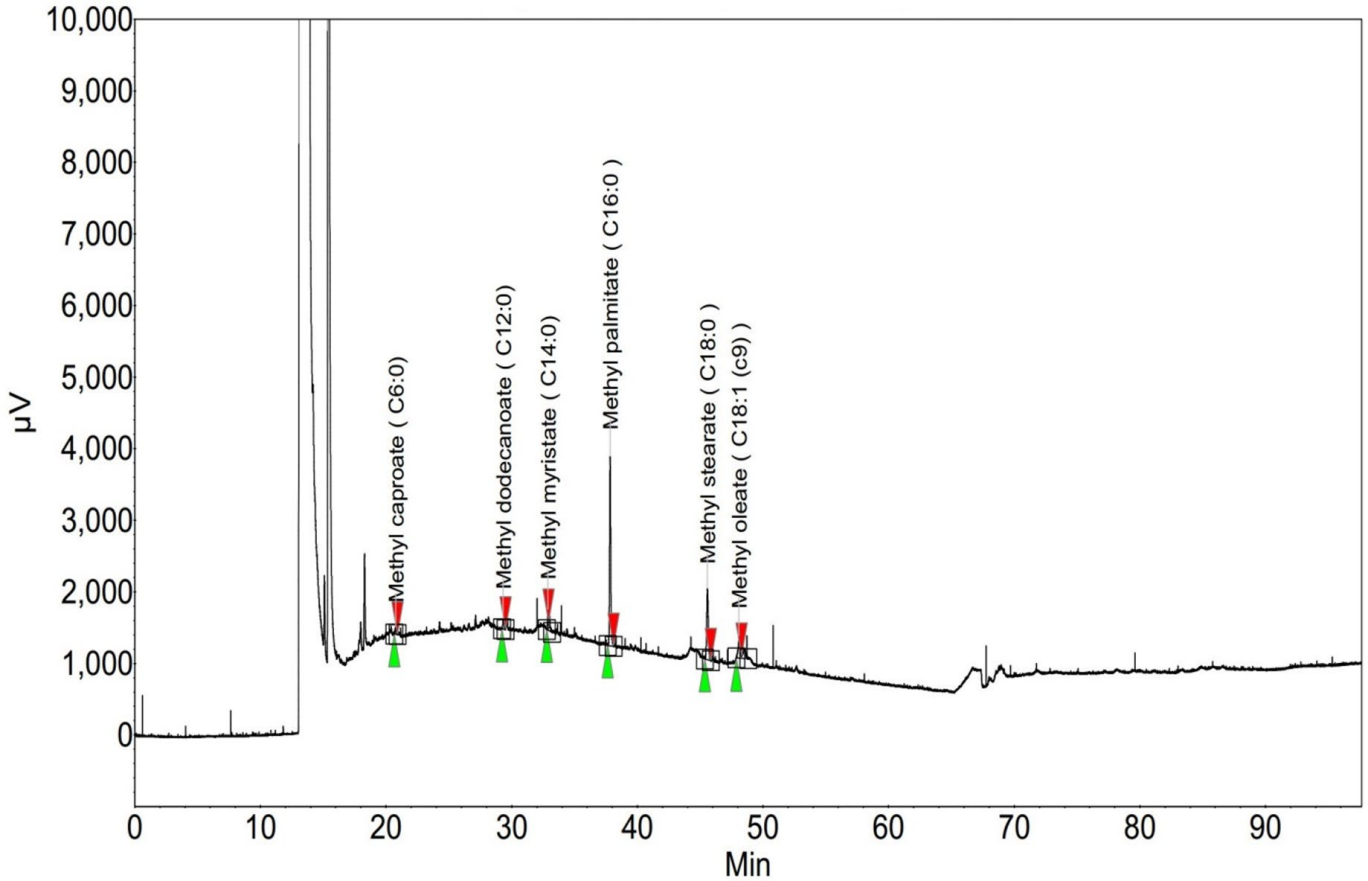
GC chromatogram of the extract of *J. rubens*.

GC analysis of fatty acid methyl esters (FAMEs) indicated that the extract contained 1.41 mg/g DW of total fatty acids, predominantly saturated fatty acids (Fig. 2; Table 1). It revealed a predominance of long-chain saturated fatty acids, mainly methyl palmitate (C16:0, 53.22%) and methyl stearate (C18:0, 27.89%), along with methyl oleate (C18:1, c9) (9.42%), which is consistent with previously reported lipid profiles of marine red algae^19^ According to Maghraby et al.^20^, the GC-MS analysis of *J. rubens* extract showed an abundance of fatty acids, e.g., myristic acid, palmitelaidic acid, palmitic acid, heptadecanoic acid, dodecanoic acid, oleic acid, stearic acid, and arachidic acid. Palmitic acid plays a crucial role in enhancing the interfacial activity of silica nanoparticles by forming hydrogen-bond-driven complexes, which improve their adsorption at the oil–water interface and significantly increase emulsion stability^21^ Stearic acid acts as a capping agent in the synthesis of silver nanoparticles, preventing agglomeration and oxidation, which leads to the formation of stable, small-sized nanoparticles with narrow size distribution^22^.

**Table 1:** GC quantitative analysis of *J. rubens* extract.

Index	Compound name	Time (min)	Quantity (mg/kg)	Height (µV)	Area (µV·min)	Area (%)
1	Methyl caproate (C6:0)	20.76	46.23	106.6	11.0	2.089
2	Methyl dodecanoate (C12:0)	29.31	53.84	150.5	18.8	3.553
3	Methyl myristate (C14:0)	32.88	53.70	293.5	20.2	3.821
4	Methyl palmitate (C16:0)	37.83	732.33	2646.8	281.0	53.224
5	Methyl stearate (C18:0)	45.58	391.09	997.1	147.2	27.891
6	Methyl oleate (C18:1, c9)	48.09	134.79	311.5	49.7	9.421

Also, oleic acid plays multiple critical roles in the synthesis of Pt nanocrystals, acting as a ligand to form a Pt-oleate complex precursor and, upon heating, generating CO that serves as a reducing agent for Pt ion reduction and nanoparticle formation^23^. Capping agents play a key role in controlling nanoparticle size, shape, stability, and aggregation by binding to the surface through functional groups such as carboxyl (-COOH), with fatty acids (saturated or unsaturated) commonly used due to their strong surface affinity and ability to enhance particle stability^24^. This type of surface coating is commonly used to inhibit particle agglomeration, enhance photocatalytic activity and stability, reduce particle dissolution, and lower potential toxicity risks^25^.

### TCC of *J. rubens* extract

The TCC of the *J. rubens* extract (Table 2) in this study was determined as 166 ± 1.05 mg GE/g DW (16.6%) using the anthrone assay, indicating a relatively high soluble carbohydrate yield. This result is consistent with the documented biochemical profile of *J. rubens* and other red macroalgae, which are generally rich in polysaccharides and other carbohydrate constituents. Previous proximate analyses of *J. rubens* from Mediterranean samples (Lebanese samples) reported significant carbohydrate fractions (14.5%) among the organic components, although the exact proportion varies with geographic location and extraction conditions^26^. Studies on red macroalgae including *J. rubens* have also demonstrated that carbohydrate content can be a major biochemical constituent, with values in some reports approaching 38.3% and 76.9% of dry weight among various Rhodophyta species, reflecting substantial soluble carbohydrate pools in these seaweeds^27^. High carbohydrate levels in seaweed extracts are particularly important due to their association with bioactive polysaccharides, such as sulfated galactans, which have been shown to exhibit antioxidant and anticancer activities *in vitro*. For example, polysaccharide fractions isolated from *J. rubens* exhibited significant antioxidant activity and cytotoxic effects against human cancer cell lines, suggesting that carbohydrate-rich extracts can contribute not only to nutritional value but also to functional biological activities^28^.

**Table 2:** Extraction yield and phytochemical composition of the *J. rubens* extract.

Parameter	Result (mg/g DW)	Percentage (%)
Total phenolic content	299.82 ± 1.83 mg GAE/g DW	29.98%
Total carbohydrate	166 ± 1.05 mg GE/g DW	16.6%
Total flavonoid content	35.61 ± 0.52 mg QE/g DW	3.561%
Total fatty acids	1.41 mg/g DW	0.141%

GAE: gallic acid equivalent; QE: quercetin equivalent; GE: glucose equivalent; DW: dry weight.

### TPC of *J. rubens* extract

The TPC of the *J. rubens* extract (Table 2) recorded the largest content and was reported as 299.82 ± 1.83 mg GAE/g DW (29.98% of dry weight). Phenolic compounds are widespread in marine macroalgae and contribute substantially to antioxidant activity through electron/hydrogen donating mechanisms. For red macroalgae such as *J. rubens*, previous studies have documented significant total phenolic levels; for example, phenolic content in *J. rubens* extracts has been reported as 24.3 ± 0.25 mg GAE/g DW algal material depending on the extraction solvent and method used, with methanol and dichloromethane/methanol extracts showing notable values^26^. Another survey of *J. rubens* from the north-eastern Mediterranean Sea also showed that total phenolics can vary considerably between species and seasons, with values reported between 34.61 and 107.0 mg GAE/g DW across brown and red species^29^.

### TFC of *J. rubens* extract

The TFC of the *J. rubens* extract (Table 2) was measured using the aluminum chloride colorimetric assay and expressed as 35.61 ± 0.52 mg QE/g DW (3.561% of dry weight). Though flavonoids are more widely studied in terrestrial plants, their presence in marine algae has been increasingly reported. In prior work on *J. rubens*, flavonoid levels have been found to vary with extraction method, with values of 28.8–42.29 mg QE/g DW reported in organic extracts of the same species^26^. These flavonoid fractions are recognized for their radical-scavenging and metal-chelating activities, which synergize with phenolic compounds to enhance overall antioxidant potential. Together, the phenolic and flavonoid contents suggest a rich phytochemical profile, consistent with reports on bioactive compounds from other red macroalgae^29^.

### Assessment of Jan-AuNPs biosynthesis by *J. rubens* hot water extract

**Fig. 3:**
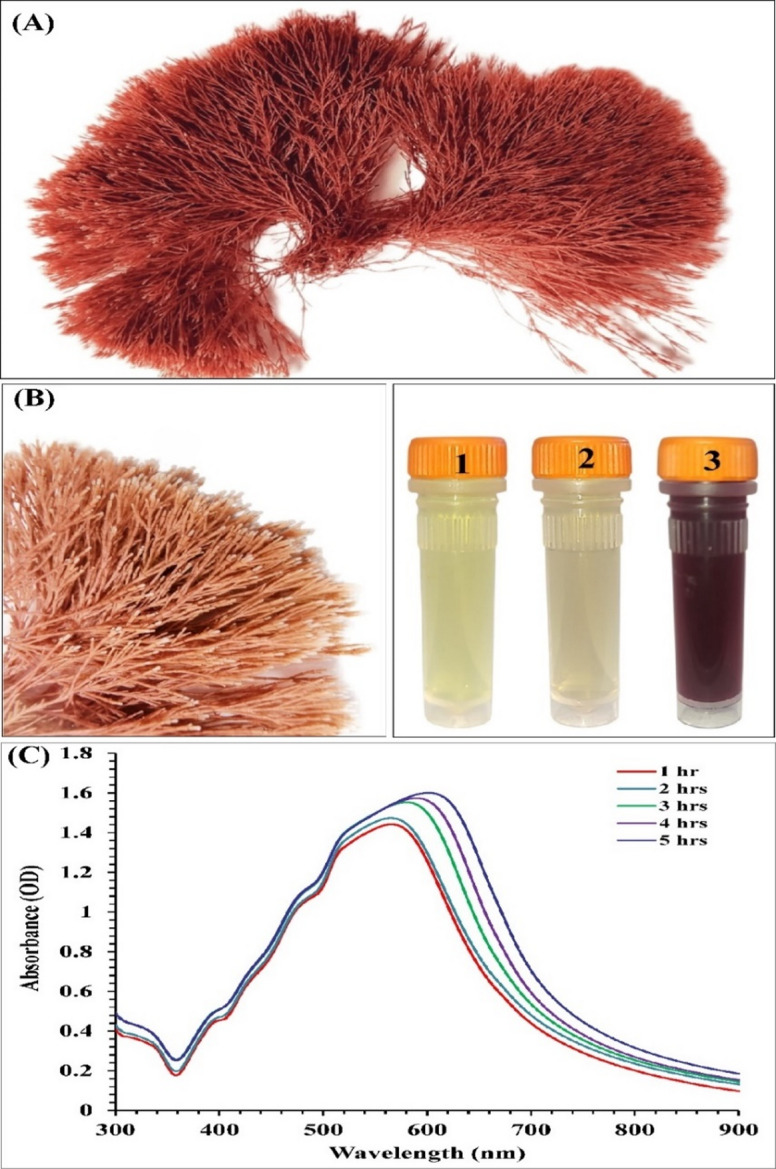
(**A,B**) Red macroalgae *J. rubens*, Vials of HAuCl_4_ solution (1), Hot water extract of *J. rubens* (2), and the biosynthesized Jan-AuNPs (3), (**C**) UV-vis spectra of Jan-AuNPs (over a period of 1–5 h).

The biosynthesis of Jan-AuNPs was effectively achieved using the hot water extract of *J. rubens* as an effective bioreductant (Fig. 3A, B). The initial visual confirmation of Jan-AuNPs synthesis was marked by a distinct color change from pale yellow to ruby red or pinkish-red, characteristic of gold nanoparticles, caused by Surface Plasmon Resonance (SPR) excitation^30^. These findings are consistent with previous studies. For instance, Kayalvizhi et al.^31^ observed increased color intensity after two days of incubation using *Turbinaria ornata* and *Padina tetrastromatica*, with *Turbinaria ornata* exhibiting a more pronounced color change. The efficient biosynthesis of AuNPs using marine algae is attributed to the reduction power of their bioactive compounds, such as polyphenols, sulfated polysaccharides, proteins, and terpenoids, that act as reducing and capping agents. These bioactive compounds reduce gold ions (Au³⁺) to elemental gold (Au⁰) and stabilize the formed nanoparticles. The reducing efficiency is positively correlated with the concentration of antioxidant-rich compounds present in the extract. El-Din and El-Ahwany^32^ reported that *J. rubens* contains a wide range of phytochemicals, including alkaloids, triterpenoids, steroids, tannins, coumarins, terpenoids, quinine, phytosteroids, phlobatannins, and flavonoids, detected in various solvent extracts. Moreover, GC-MS analysis of *J. rubens* crude extracts revealed numerous constituents, with ascorbic acid being among the main constituents detected in high percentages. As noted by Malassis et al.^33^, ascorbic acid plays a pivotal role in the biosynthesis of AuNPs, enabling the formation of stable nanoparticles with controlled size distribution ranging from 8 to 80 nm.

Rifi et al.^26^ reported that proximate analysis of *J. rubens* powders revealed comparatively higher levels of proteins and carbohydrates than lipids. Biomolecules such as proteins, carbohydrates, alkaloids, phenols, saponins, and flavonoids play a significant role in the biosynthesis of gold nanoparticles by reducing gold ions (Au³⁺) to elemental gold (Au⁰) and stabilizing the resulting nanoparticles, owing to their potent reducing power and capping abilities^34^. According to Ahmed et al.^35^, gas–liquid chromatography (GLC) analysis of fatty acid methyl esters revealed that the main unsaturated fatty acid in *J. rubens* was oleic acid, whereas the most predominant saturated fatty acid was palmitic acid, followed by myristic acid. As outlined by Kuppusamy et al.^36^, the biosynthesis of gold nanoparticles from their metal salt precursors typically involves various constituents, including reducing sugars, polyphenols, proteins, alkaloids, and flavonoids, which act as both reducing and capping agents. In this context, key fatty acids such as oleic and palmitic acids present in the water-soluble extract of *J. rubens* may serve as efficient capping agents, enhancing nanoparticles’ stability and preventing aggregation.

### UV–Vis spectral analysis of Jan-AuNPs

UV–Visible (UV–Vis) spectroscopy is one of the most widely employed techniques for characterizing the optical properties of gold nanoparticles, as it is highly sensitive to particle size, shape, concentration, and colloidal stability. Spectral analysis was conducted in the wavelength range of 300–800 nm to verify the formation of Jan-AuNPs. A prominent SPR absorption peak was detected at 549 nm, indicating the successful reduction of HAuCl₄ ions and formation of Jan-AuNPs. Over time, the absorption maxima increased in intensity and shifted from 565 to 601 nm with extended incubation, as shown in Fig. 3C. The broadening of the UV–Vis absorption band can be attributed to the presence of biomolecules (such as proteins, polysaccharides, phenolic compounds, and pigments) derived from the *J. rubens* biomass extract used during biosynthesis. These biomolecules can adsorb onto the nanoparticles surface, acting as natural capping agents. Their functional groups (e.g., –OH, –COOH, –NH₂) can electronically interact with the nanoparticle’s surface electrons. As a result, the UV–Vis absorption band becomes broader or slightly shifted, reflecting changes in the surface chemistry of the nanoparticles. Namvar et al.^37^ reported an absorption peak at 550 nm during the synthesis of AuNPs by *Sargassum muticum*. Similarly, Rosyidah et al.^38^ detected an SPR band at 552 nm in nanoparticles synthesized using *Synechococcus moorigangae*. In another study, Gürsoy et al.^39^ observed an initial SPR peak at 547 nm after 24 h of incubation with *Chlorella sorokiniana*, which shifted to 554 nm after 48 h, indicating enhanced nanoparticles’ formation. Additionally, *Turbinaria conoides* showed a weak SPR band at 536 nm, which slightly shifted to 540 nm after 90 min, likely due to variations in nanoparticles’ size and morphology^40^.

### Microscopy analysis of Jan-AuNPs

**Fig. 4:**
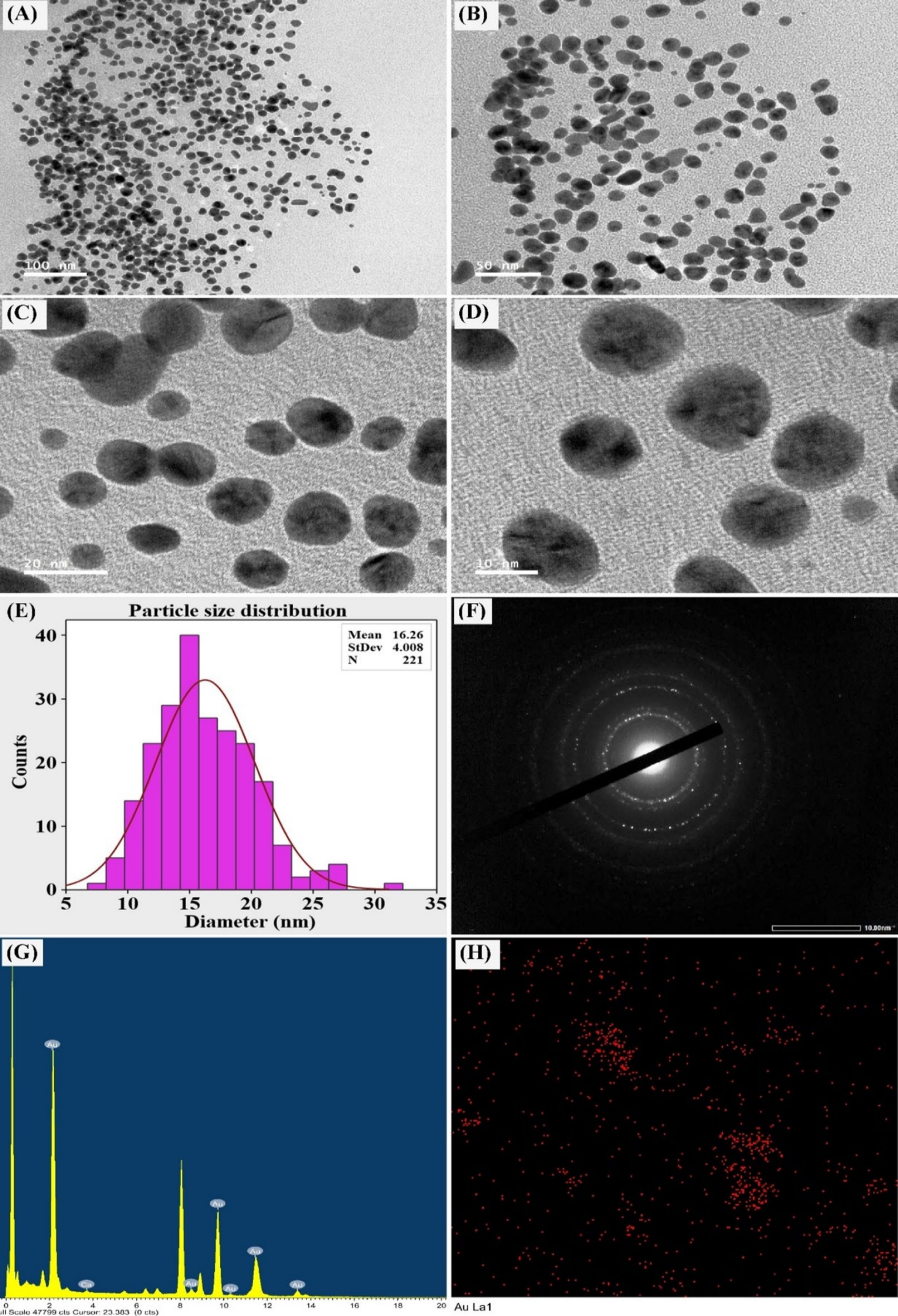
TEM images of biosynthesized Jan-AuNPs at 4 different nano scale bars; 100 nm scale (**A**), at 50 nm scale (**B**), at 20 nm scale (**C**) and at 10 nm scale (**D**), particle size distribution (**E**), SAED pattern (**F**), EDX analysis (**G**) and mapping analysis (**H**) of biosynthesized Jan-AuNPs.

Transmission electron microscopy (TEM) was employed to examine the purity, polydispersity, surface morphology, size, and shape of the biosynthesized Jan-AuNPs (Fig. 4A-D). The TEM images confirmed successful biosynthesis, revealing that they were well-dispersed, relatively uniform, and predominantly spherical particles, with some rod-shaped particles also observed. The average particle diameter was measured at 16.26 nm (Fig. 4E). These findings are in line with previous studies. Emami et al.^41^ described the nanoparticles synthesized in their study as almost spherical with faceted surfaces, with an average particle diameter of 16 nm. Additionally, Kamal et al.^42^ observed primarily spherical gold nanoparticles with diameters between 9.3 and 22 nm (average 15.5 nm) synthesized from *Cystoseira trinodis* extracts. The size distribution obtained in this study appears more favorable compared to the findings of Mikhailova^43^, who reported gold nanoparticles synthesized from *Sargassum polycystum* with diameters ranging from 64 to 240 nm. In agreement with our results, Rajathi et al.^44^ analyzed AuNPs synthesized using *Stoechospermum marginatum* biomass and reported predominantly spherical nanoparticles, along with hexagonal and triangular shapes, with sizes between 18.7 and 93.7 nm. Similarly, Oza et al.^45^ found that AuNPs synthesized by the freshwater algae *Chlorella pyrenoidosa* ranged from 25 to 30 nm. Ramakrishna et al.^40^ also reported a size range of 12 to 57 nm, with an average of 27.5 nm, for nanoparticles synthesized using *Turbinaria conoides*.

The crystallinity of the biosynthesized Jan-AuNPs was evaluated using selected area electron diffraction (SAED) patterns obtained through TEM analysis. The SAED pattern (Fig. 4F) exhibited well-defined circular rings with distinct bright spots dispersed along the rings, confirming the polycrystalline nature of the Jan-AuNPs. These diffraction rings correspond to the Bragg reflection planes (111), (200), (220), and (311), which are characteristic of the face-centered cubic (fcc) crystalline structure of gold nanoparticles^46^. Similar observations were reported by Senthilkumar et al.^47^, who found that the SAED pattern of AuNPs synthesized using *Gelidiella acerosa* exhibited a polycrystalline structure. In contrast, Babu et al.^48^ reported that AuNPs biosynthesized from *Acanthophora spicifera* displayed single crystallinity with concentric fcc phase rings corresponding to the (111), (220), and (311) lattice planes of gold.

The elemental composition of the biosynthesized Jan-AuNPs was determined using energy-dispersive X-ray spectroscopy (EDX), as shown in Fig. 4G. The EDX spectrum confirmed a strong signal for elemental gold, with a weight% of 99.62%, validating the successful synthesis and high purity of Jan-AuNPs. The most intense gold signal appeared in the 2–3 keV energy range, which is characteristic of gold and consistent with the findings of Babu et al.^48^, who reported a similar EDX peak for AuNPs synthesized using *Acanthophora spicifera*. Additionally, a minor calcium signal was also detected with a weight% of 0.38%, due to the presence of high levels of calcium carbonate in *J. rubens*, which contributes to its hard texture. In comparison, Correa et al.^49^ reported that gold nanoparticles comprised 12.88% of the total elemental composition in their sample, as confirmed by EDX analysis.

Elemental mapping analysis was conducted to visualize the spatial distribution of elements within the biosynthesized Jan-AuNPs. The mapping results confirmed a uniform and dense distribution of gold atoms within the Jan-AuNPs (Fig. 4H). This homogeneity and dense distribution of gold signals in the mapped image indicate efficient reduction and stabilization of gold ions by the *J. rubens* extract and consistent synthesis of Jan-AuNPs.

### FTIR analysis of biosynthesized Jan-AuNPs

**Fig. 5:**
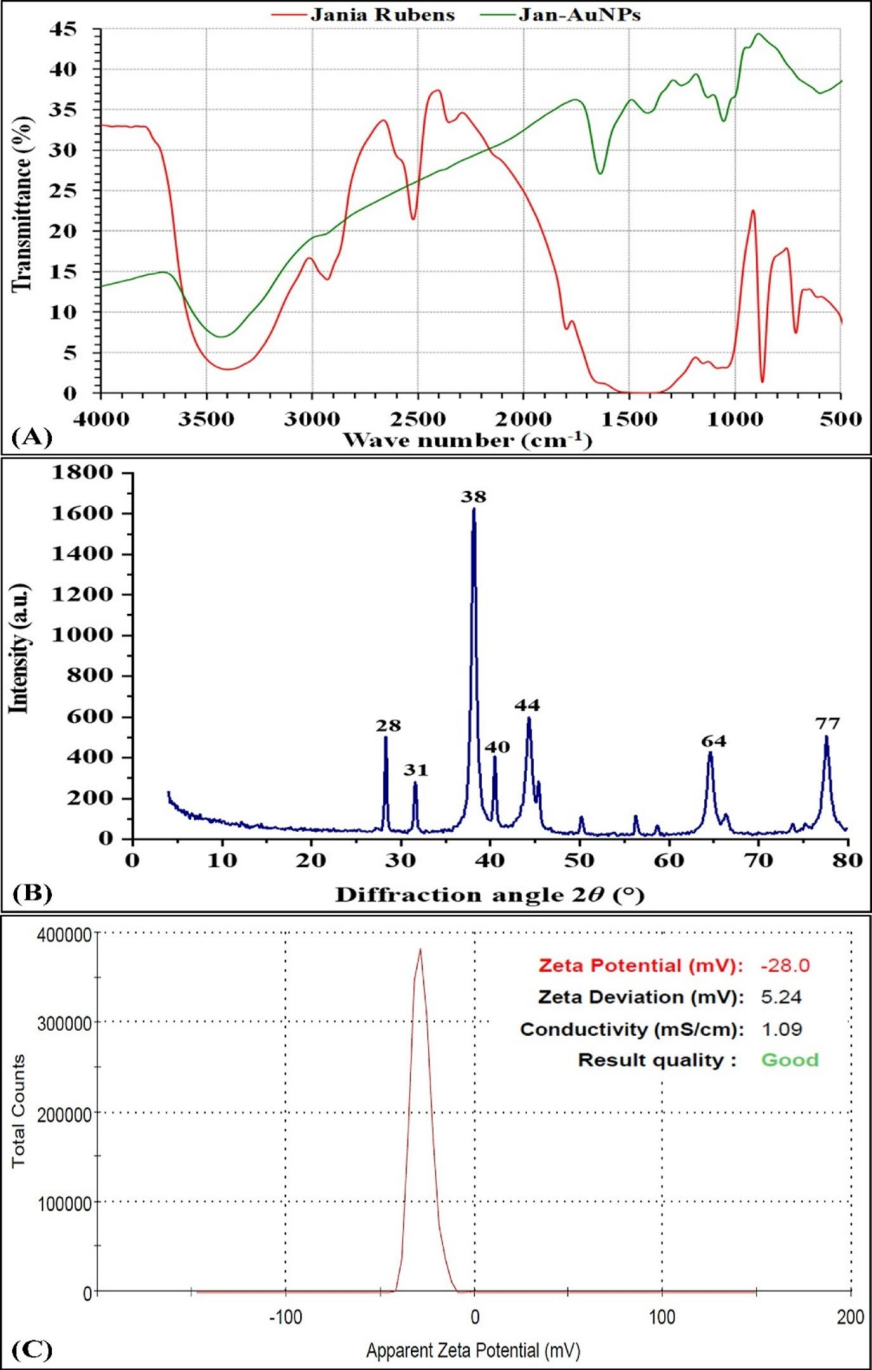
FTIR analysis of the hot water extract of *J. rubens* and biosynthesized Jan-AuNPs (**A**), XRD (**B**), and ζ-potential (**C**) of biosynthesized Jan-AuNPs.

Fourier-transform infrared (FTIR) spectroscopy was employed to identify the functional groups present in the *J. rubens* extract that are involved in the reduction and stabilization of gold ions during the green synthesis process. The FTIR spectra of the *J. rubens* extract and the biosynthesized Jan-AuNPs using *J. rubens* show clear spectral differences, reflecting chemical changes due to nanoparticles’ formation and interaction with biomolecules (Fig. 5A). The FTIR spectroscopy of the obtained Jan-AuNPs showed 8 absorption peaks at 3431, 1637, 1414, 1254, 1129, 1055, 938, and 597 cm^− 1^. In contrast, the FTIR spectroscopy of the algal extract of *J. rubens* showed 9 sharp absorption peaks at 3400, 2929, 2522, 2353,1797, 1419,1045, 871, and 713 cm^− 1^. The O–H stretching band observed at 3431 cm^− 1^ in Jan-AuNPs and at 3400 cm^− 1^ in the *J. rubens* extract indicates the presence of hydroxyl groups, likely derived from alcohols or phenols^50^. The slight shift between these bands suggests active participation of hydroxyl groups in both the reduction of Au³⁺ ions and Jan-AuNPs stabilization. A prominent peak at 2929 cm^− 1^ in the *J. rubens* spectrum is attributed to aliphatic C–H stretching vibrations, specifically from methylene (–CH_2_–) groups in long carbon chains or fatty acids^51^. Additionally, a distinct peak at 2522 cm⁻¹ corresponds to S–H (thiol) stretching vibrations, indicating the presence of thiol groups that may contribute to the bioreduction of Au^3+^ and enhance Jan-AuNPs stability^52^.

A band observed at 2353 cm^− 1^ in the *J. rubens* extract may be attributed to nitrile (C ≡ N) or alkyne (C ≡ C) groups, commonly found in carboxylic acids or other organic acids^53^. The carbonyl (C = O) groups are evidenced by bands at 1797 cm^− 1^ in the extract and 1637 cm^− 1^ in Jan-AuNPs, likely originating from fatty acids that play roles in nanoparticles’ stabilization and surface capping. The band at 1419 cm⁻¹ in the *J. rubens* extract and its slight shift to 1414 cm^− 1^ in Jan-AuNPs are indicative of C–O stretching in carboxylic acids or deformation vibrations of –C–OH groups in polysaccharides^53^. This shift suggests that these functional groups interacted with gold ions during nanoparticles’ formation, contributing to both reduction and capping. The binding of these groups to the nanoparticles’ surface alters their chemical environment, resulting in peak shifts. In Jan-AuNPs, a broad band between 1254 and 1055 cm^− 1^ includes signals corresponding to C = O and C–O stretching vibrations, typically associated with carboxylic acid (–COOH) groups from organic acids or esters, which are implicated in reduction and stabilization processes^54^. A distinct peak at 1129 cm^− 1^ is attributed to C–O–C stretching vibrations, often linked to fatty acid esters or ether linkages, suggesting the involvement of lipid-derived compounds in the algal extract^55^. Additional bands at 1055 and 1076 cm^− 1^ correspond to C–N stretching vibrations of aliphatic amines, which are also known to aid in gold nanoparticles capping and stabilization^56^. A peak at 938 cm^− 1^ is assigned to O–H in-plane and out-of-plane bending, highlightin5 the role of hydroxyl-containing compounds in the synthesis and stability of Jan-AuNPs^57^. Furthermore, the absorption band at 597 cm^− 1^ is associated with Cu–O stretching vibrations, possibly indicating minor contamination with copper oxide nanoparticles^58^. Weak bands observed in the 529–555 cm^− 1^ region may be attributed to coordination interactions between metal ions and water or phenolic compounds, potentially involved in metal chelation during the biosynthesis process.

### XRD analysis of biosynthesized Jan-AuNPs

The crystalline nature and compositions of the purified Jan-AuNPs synthesized using *J. rubens* extract were confirmed by X-ray diffraction (XRD) analysis, as shown in Fig. 5B. Distinct diffraction peaks were observed at 2θ values of 2*θ* = 28°, 31°, 38°, 40°, 44°, 64°, and 77°. Among these, the peaks at 38°, 44°, 64°, and 77° correspond to the (111), (200), (220), and (311) planes of the face-centered cubic (fcc) crystalline structure of gold. These reflections confirm the formation of crystalline AuNPs. In contrast, the peaks observed at 28°, 31°, and 40° may be attributed to amorphous components, possibly originating from the biomolecules in the *J. rubens* extract that act as capping and stabilizing agents. These findings are consistent with previous studies.

Singh et al.^59^ reported that AuNPs synthesized using *Padina gymnospora* exhibited Bragg reflections at 2*θ* values of 38.11°, 44.25°, 64.08°, and 77.5°, corresponding to the (111), (200), (220), and (311) planes of fcc gold. Similarly, Rosyidah et al.^38^ identified four prominent diffraction peaks at 38.12°, 44.31°, 64.42°, and 78.57°, which aligned to the (111), (200), (220), and (311) planes of fcc crystalline gold. Furthermore, El-Naaggr et al.^8^ confirmed the crystalline nature of gold nanoparticles with a strong diffraction peak at a 2*θ* angle of 38.2°, indicating a preferential growth orientation along the (111) plane.

### Zeta potential (ζ) analysis of biosynthesized Jan-AuNPs

Zeta potential analysis is a crucial method for evaluating the surface charge and colloidal stability of nanoparticles, as it reflects the degree of electrostatic repulsion or attraction between particles, which affects the stability of the gold nanoparticles^60^. The zeta potential of the biosynthesized Jan-AuNPs was recorded at − 28 mV (Fig. 5C), indicating a strong negative surface charge. A high absolute zeta potential value suggests a significant electrostatic repulsion between particles, which prevents aggregation and supports the long-term colloidal stability of the nanoparticles^61^. The large negative charge helps to stabilize the Jan-AuNPs by inhibiting particle–particle aggregation, thereby maintaining a well-dispersed colloidal system^62^. This negative surface charge likely originates from the anionic functional groups (e.g., carboxylates, hydroxyls, and thiols identified in the FTIR spectrum), which are present in the algal biomolecules and act as capping agents during the biosynthesis process. Comparable findings have been reported in the literature. According to Hatipoğlu^63^, the average zeta potential of gold nanoparticles was reported to be − 27 mV. Similarly, Rosyidah et al.^38^ demonstrated that the zeta potential of biosynthesized gold nanoparticles was − 21.5 mV, indicating moderate colloidal stability. Furthermore, Kamal et al.^42^ reported zeta potential values of − 20 mV, − 40.3 mV, and − 47.2 mV for gold nanoparticles synthesized using *Cystoseira myrica*, *C. trinodis*, and *C. prolifera*, respectively, highlighting the influence of algal species on nanoparticles’ surface charge and stability.

### Optimization of Jan-AuNPs biosynthesis by face-centered central composite design (FCCCD)

The FCCCD is a statistical approach within Response Surface Methodology (RSM) that serves as an effective tool for exploring and optimizing multiple experimental parameters and their interactions. It is particularly effective for optimizing biosynthetic processes, such as the biosynthesis of Jan-AuNPs. By applying FCCCD, the effects of critical factors, such as initial pH, temperature, gold ion concentration, and incubation time on the biosynthesis of Jan-AuNPs can be thoroughly assessed. This method not only identifies optimal conditions for maximum Jan-AuNPs yield but also provides valuable insights into key variable interactions, ultimately enhancing the stability, efficiency, and reproducibility of the biosynthesis process. Preliminary experiments were conducted to assess the stability of the biosynthesized Jan-AuNPs under different pH conditions prior to optimization using FCCCD. The nanoparticles exhibited good stability and dispersion within the pH range of 6–8. However, at pH 9, the solution color changed from pinkish-red to dark violet, accompanied by visible aggregation and precipitation, indicating a reduction in colloidal stability under alkaline conditions.

**Table 3:** FCCCD matrix for Jan-AuNPs biosynthesis by *J. rubens* showing coded and actual levels of pH, temperature, incubation time, and gold concentration.

Std	Run	A	B	C	D	Jan-AuNPs biosynthesis (µg/mL)					Residuals
						Actual		Predicted			
23	1	0	0	0	-1	117		120.15			-3.15
13	2	-1	-1	1	1	205		203.44			1.56
14	3	1	-1	1	1	216		222.40			-6.40
17	4	-1	0	0	0	140		146.04			-6.04
8	5	1	1	1	-1	78		80.68			-2.68
2	6	1	-1	-1	-1	64		68.62			-4.62
1	7	-1	-1	-1	-1	55		48.66			6.34
4	8	1	1	-1	-1	72		67.88			4.12
20	9	0	1	0	0	179		177.15			1.85
22	10	0	0	1	0	187		177.04			9.96
12	11	1	1	-1	1	219		224.62			-5.62
28	12	0	0	0	0	192		185.80			6.20
16	13	1	1	1	1	240		240.66			-0.66
3	14	-1	1	-1	-1	60		60.18			-0.18
7	15	-1	1	1	-1	71		70.72			0.28
11	16	-1	1	-1	1	225		220.16			4.84
30	17	0	0	0	0	181		185.80			-4.80
21	18	0	0	-1	0	169		175.37			-6.37
9	19	-1	-1	-1	1	209		212.90			-3.90
10	20	1	-1	-1	1	235		229.61			5.39
19	21	0	-1	0	0	164		162.26			1.74
6	22	1	-1	1	-1	59		58.16			0.84
29	23	0	0	0	0	181		185.80			-4.80
24	24	0	0	0	1	289		282.26			6.74
26	25	0	0	0	0	186		185.80			0.20
25	26	0	0	0	0	180		185.80			-5.80
18	27	1	0	0	0	169		159.37			9.63
27	28	0	0	0	0	184		185.80			-1.80
15	29	-1	1	1	1	232		233.95			-1.95
5	30	-1	-1	1	-1	35		35.95			-0.95
Variable	Code	-1	0	1							
Initial pH level	A	6	7	8							
Temperature (ºC)	B	40	60	80							
Incubation time (hrs)	C	1	3	5							
Gold concentration (µg/mL)	D	100	200	300							

For the optimization of Jan-AuNPs biosynthesis, Table 3 summarizes both the actual and predicted responses obtained through the FCCCD model. The table includes four independent variables: A (initial pH level), B (temperature), C (incubation time), and D (gold ion concentration), each evaluated at three coded levels (-1, 0, and 1). A total of 30 experimental runs were conducted to explore the optimal combination of these variables. The highest yield of Jan-AuNPs (289 µg/mL) was observed in run number 24 under the conditions of pH 7, temperature 60 °C, incubation time of 3 h, and gold ion concentration of 300 µg/mL. Conversely, the lowest yield (35 µg/mL) was recorded in run number 30 at pH 6, temperature 40 °C, incubation time of 5 h, and gold concentration of 100 µg/mL.

### ANOVA and multiple regression analysis

Analysis of variance (ANOVA) was employed to statistically evaluate the biosynthesis of Jan-AuNPs, as presented in Table 4. Key statistical parameters, including the *F*-value (Fisher’s statistic), *P*-value (probability), lack of fit, R² (coefficient of determination), predicted R², adjusted R², sum of squares, and mean squares, were analyzed. As well as the linear, interaction, and quadratic effects of the four independent variables (A, B, C, and D) were examined to assess the reliability and significance of the model^64^. The statistical significance of the model and its individual variables and their interactions was primarily determined using *P*-values. A *P*-value less than 0.05 typically indicates a statistically significant effect^65^. The FCCCD model demonstrated high statistical significance, with an *F*-value of 209.06 and a *P*-value < 0.0001, confirming its robustness and predictive capacity.

Among the independent variables, initial pH (A), temperature (B), and gold concentration (D) had highly significant linear effects on Jan-AuNPs biosynthesis. Their low *P*-values (0.0008, 0.0003, and < 0.0001, respectively) and high *F*-values (17.48, 21.80, and 2583.86) demonstrated their significant linear impacts on Jan-AuNPs biosynthesis. In contrast, incubation time (C) did not exert a significant linear effect, as indicated by its high *P*-value (0.6089) and low *F*-value (0.27). Positive coefficients for pH (6.67), temperature (7.44), and gold concentration (81.06) suggest that increasing these variables promotes Jan-AuNPs biosynthesis. Among them, gold concentration demonstrated the strongest effect. In contrast, the incubation time had no significant effect. Regarding interaction effects, only the BC interaction (temperature × incubation time) was statistically significant, with a *P*-value of 0.0037, an *F*-value of 11.81, and a positive coefficient of 5.81. This indicates that higher temperatures coupled with longer incubation periods synergistically enhance Jan-AuNPs biosynthesis. Other interactions (AB, AC, AD, BD, and CD) showed no significant effects, as reflected by their *P*-values > 0.05. The quadratic effects provided further insights into the non-linear influence of each variable on Jan-AuNPs biosynthesis. For initial pH (A²), the negative coefficient (-33.10), high *F*-value (62.01), and highly significant *P*-value (< 0.0001) indicate that the biosynthesis increases up to an optimal pH level and subsequently declines. A similar trend was observed for temperature (B²), with a negative coefficient (-16.10), an *F*-value of 14.67, and a significant *P*-value of 0.0016, confirming that extreme temperatures lead to a decline in Jan-AuNPs biosynthesis once the optimal value is exceeded. The quadratic effect of incubation time (C²) also displayed a negative coefficient (-9.60), with statistical significance (*F* = 5.21, *P* = 0.0374), suggesting a similar peak-and-decline trend. In contrast, the quadratic term for gold ion concentration (D²) exhibited a positive coefficient (15.40), an *F*-value of 13.43, and a significant *P*-value (0.0023), indicating that, unlike other variables, gold concentration exhibited a quadratic trend with a positive coefficient, indicating improved biosynthesis as levels increased within the tested range.

**Table 4:** ANOVA for Jan-AuNPs biosynthesis by *J. rubens* as influenced by pH, temperature, incubation time, and gold concentration.

Source of variance		Coefficient estimate	Sum of squares	Degrees of freedom	Mean square	F-value	*P*-value
Intercept	Intercept	185.80	134000.00	14	9568.63	209.06	< 0.0001
Linear effect	A	6.67	800.00	1	800.00	17.48	0.0008
	B	7.44	997.56	1	997.56	21.80	0.0003
	C	0.83	12.50	1	12.50	0.27	0.6089
	D	81.06	118300.00	1	118300.00	2583.86	< 0.0001
Interaction effect	AB	-3.06	150.06	1	150.06	3.28	0.0903
	AC	0.56	5.06	1	5.06	0.11	0.7441
	AD	-0.81	10.56	1	10.56	0.23	0.6379
	BC	5.81	540.56	1	540.56	11.81	0.0037
	BD	-1.06	18.06	1	18.06	0.39	0.5393
	CD	0.81	10.56	1	10.56	0.23	0.6379
Quadratic effect	A²	-33.10	2838.02	1	2838.02	62.01	< 0.0001
	B²	-16.10	671.30	1	671.30	14.67	0.0016
	C²	-9.60	238.60	1	238.60	5.21	0.0374
	D²	15.40	614.74	1	614.74	13.43	0.0023
Error effect	Lack of fit		584.53	10	58.45	2.87	0.13
	Pure error		102.00	5	20.40		
R^2^	0.9949	Std. dev.	6.77				
Adj R^2^	0.9901	Mean	159.77				
Pred R^2^	0.9747	C.V. %	4.23				
Adeq precision	51.49	PRESS	3408.19				

* C.V: Coefficient of variation, *P*: Level of significance, *F*: Fisher’s function.

The adequacy of the model and the proportion of variation in the variable that can be attributed to the independent variables and their interactions can be assessed through the coefficient of determination, R². When this value exceeds 0.9, the regression model is regarded as demonstrating a very strong correlation^66,67^. In this study, the model exhibited a high R² value of 0.9949, suggesting that 99.49% of the variation in Jan-AuNPs biosynthesis was explained by the model, leaving only 0.51% unexplained. The adjusted R² (Adj R²) reflects the proportion of variance in the response explained by the model as influenced by the independent factors, while the predicted R² (Pred R²) is utilized to assess the model’s ability to predict response values across different levels of the examined process variables in subsequent experiments^68^. A strong agreement between the Adj R² (0.9901) and Pred R² (0.9747) confirms the model’s robustness and strong predictive performance. An Adequate Precision value of 51.49 further confirms the robustness and reliability of the model. A ratio greater than 4 is generally considered desirable, indicating that the model can effectively differentiate between signal (true model response) and noise (random error). In this case, the high Adequate Precision value of 51.49 indicates a very strong signal relative to noise, confirming that the model provides highly reliable predictions and is suitable for optimizing the experimental conditions. The predicted residual sum of squares (PRESS) was 3408.19, and the coefficient of variation (C.V.) was relatively low at 4.23%, suggesting high precision of the experimental results^69^. The model also reported a mean response value of 159.77 with a standard deviation of 6.77, further supporting its robustness and reliability.

The interaction between independent and dependent variables was studied by applying a second-order polynomial equation. Using the levels of pH, temperature (°C), incubation period (h), and gold concentration (µg/mL), the second-order polynomial equation was utilized to estimate the maximum Jan-AuNPs biosynthesis.

Jan-AuNPs biosynthesis (µg/mL) = + 185.80 + 6.67 * pH + 7.44 * temperature + 0.83* incubation period + 81.06 * gold concentration − 3.06 * pH * temperature + 0.56 * pH * incubation period − 0.81 * pH * gold concentration + 5.81 * temperature * incubation period − 1.06 * temperature * gold concentration + 0.81 * incubation period * gold concentration − 33.10 * pH² -16.10* temperature ² -9.60 * incubation period ² + 15.40 * gold concentration ².

### Fit summary analysis

Table 5 illustrates the FCCCD fit summary results of the biosynthesis of Jan-AuNPs using *J. rubens* hot water extract. The fit summary was utilized to select the efficient model from linear, two-variable interactions (2FI) and quadratic models, based on the statistical significance of each model type in predicting and maximizing Jan-AuNPs biosynthesis. The results clearly indicate that the quadratic model is the most suitable for describing and predicting Jan-AuNPs biosynthesis. This is strongly supported by its high coefficient of determination (R² = 0.9949), along with an adjusted R² of 0.9901 and a predicted R² of 0.9747. Additionally, the model demonstrated minimal prediction error, as indicated by a low PRESS value (3408.19) and a low standard deviation (6.77), further confirming its robustness. Moreover, the lack-of-fit test for the quadratic model was not statistically significant (*P* = 0.1284), suggesting that the model fits the experimental data well.

### Three-dimensional (3D) plot

**Fig. 6:**
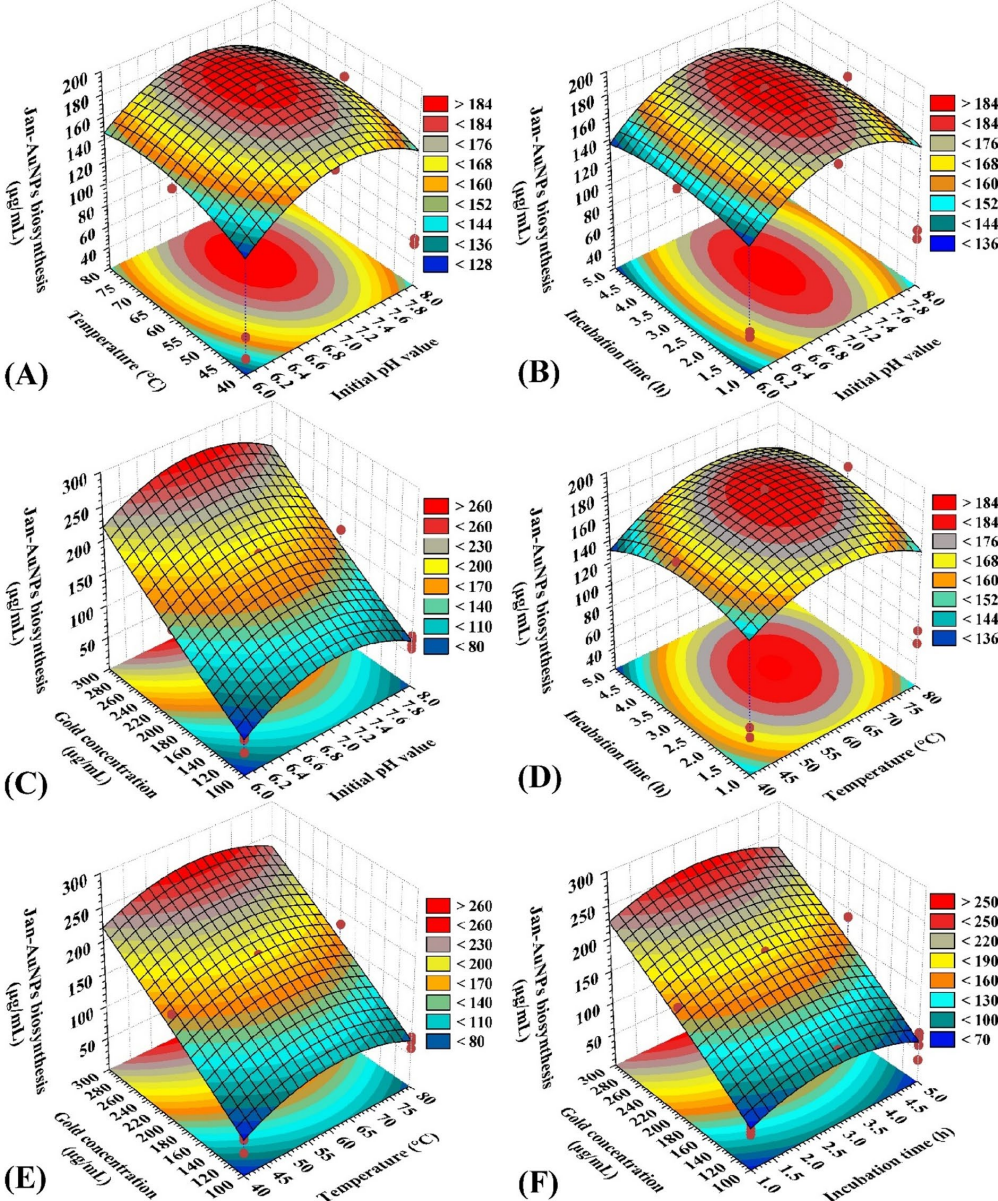
3D plots showing the mutual effects of initial pH level, temperature, incubation time, and gold concentration on Jan-AuNPs biosynthesis by *J. rubens*.

The 3D response surface plots (Fig. 6) illustrate the interactive effects of initial pH, temperature, incubation time, and gold ion concentration on the biosynthesis of Jan-AuNPs using *J. rubens* extract. Each subfigure (Fig. 6A–F) demonstrates the combined influence of two independent variables on the biosynthesis of Jan-AuNPs, while the remaining two variables are held constant at their central (zero-coded) levels.

**Table 5:** Fit summary for Jan-AuNPs biosynthesis by *J. rubens* showing effects of pH, temperature, incubation time, and gold concentration.

Source	Sum of squares	Df	Mean square	F-value	*P*-value
**Lack of fit tests**					
Linear	14475.26	20	723.76	35.48	0.0004*
2FI	13740.38	14	981.46	48.11	0.0002*
Quadratic	584.53	10	58.45	2.87	0.1284
**Sequential model sum of squares**					
Linear vs. Mean	120100.00	4	30017.53	51.48	< 0.0001*
2FI vs. Linear	734.88	6	122.48	0.17	0.9823
Quadratic vs. 2FI	13155.85	4	3288.96	71.86	< 0.0001*
**Model summary statistics**					
Source	Standard deviation	R^2^	Adjusted R^2^	Predicted R^2^	PRESS
Linear	24.15	0.8917	0.8744	0.8469	20619.06
2FI	26.99	0.8972	0.8431	0.6444	47886.17
Quadratic	6.77	0.9949	0.9901	0.9747	3408.19
**Fit summary**					
Source	Sequential *P-*value	Lack of fit *P-*value	Adjusted R²	Predicted R²	
Linear	< 0.0001*	0.0004	0.8744	0.8469	
2FI	0.9823	0.0002	0.8431	0.6444	
Quadratic	< 0.0001*	0.1284	0.9901	0.9747	Suggested

* Significant values, *df*: degree of freedom, PRESS: sum of squares of prediction error, 2FI: two variables interaction.

### Effect of initial pH on biosynthesized Jan-AuNPs

The initial pH value was found to be a critical factor influencing the biosynthesis yield of Jan-AuNPs. The yield of Jan-AuNPs was highly sensitive to pH variations, highlighting the importance of pH in modulating the biochemical environment required for reduction and capping. The highest biosynthesis was consistently achieved around neutral to slightly alkaline pH (6.8–7.4), where the ionization state of the biomolecules in *J. rubens* (such as phenolics and proteins) is in their most active form for electron transfer, maximizing their reducing and capping ability. In contrast, at lower or higher pH levels, the biosynthetic capacity of Jan-AuNPs declined, which could be attributed to the precipitation of metal ions or conformational changes in the reducing biomolecules.

Initial pH and temperature showed a significant synergistic effect on Jan-AuNPs biosynthesis (Fig. 6A). Maximum Jan-AuNPs biosynthesis yield (> 184 µg/mL) was obtained at a moderately acidic to neutral pH (6.5–7.0) and temperatures around 50–55 °C. Under these conditions, the reducing and stabilizing biomolecules in the *J. rubens* extract are most active and thermally stable. Outside these optimal levels, either at extreme pH or temperature values, a steep decline in Jan-AuNPs biosynthesis yield was recorded. Low pH values (< 5.5) inhibited biosynthesis, likely due to the denaturation of functional groups on the biomolecules participating in Jan-AuNPs synthesis, reducing their availability for metal ion reduction. High temperature levels led to thermal degradation of biomolecules in the *J. rubens* extract, decreasing the reduction and stabilization of Jan-AuNPs.

The response surface plots reveal the interaction between pH and incubation time (Fig. 6B). The optimal pH remained around 6.5–7.5, with maximal Jan-AuNPs biosynthesis observed after approximately 2.5–3 h of incubation. The response surface revealed that an incubation period of approximately 2.5–3 h at pH 6.5–7.5 represents the optimal incubation period, resulting in maximum Jan-AuNPs biosynthesis. Short incubation times (< 1.5 h) yielded low Jan-AuNPs regardless of pH, suggesting that time is required for complete reduction. Prolonged incubation periods (> 4 h) resulted in a decline in yield, due to aggregation of Jan-AuNPs or degradation of biomolecules. The combined effect of pH and gold ion concentration (100–300 µg/mL) revealed that biosynthesis yield increased significantly with higher gold ion concentrations, particularly at pH 7–7.5 (Fig. 6C). Yields exceeding 260 µg/mL were observed when the gold concentration exceeded 250 µg/mL. This can be attributed to the enhanced reactivity of functional groups in this pH range, optimizing reduction efficiency. At pH values outside the ideal pH range, a marked decline was noted, possibly due to inactivation of reducing agents.

These findings align with previous studies highlighting the critical role of pH in the biosynthesis of AuNPs. Matta et al.^70^ reported that the brown alga *Fucus vesiculosus* exhibited the highest reduction of gold ions at pH 7, with effective activity across a broader pH range of 4 to 9. Similarly, Gürsoy et al.^39^ evaluated the effect of pH variation on AuNPs biosynthesis by *Chlorella sorokiniana* and found that pH 8 yielded the highest nanoparticles’ formation. Neutral to slightly alkaline conditions are often favorable due to their stabilizing effects on the interaction between gold ions and surface-bound proteins, particularly amino groups^71^. Consistently, Oza et al.^45^ demonstrated that pH 8 was optimal for producing stable AuNPs using *Chlorella pyrenoidosa* extract, while lower pH values (2 and 4) led to larger, more aggregated particles. Ariski et al.^72^ further observed that during the synthesis of AuNPs from *Heju Hallabong* peel extract, an increase in pH correlated with the formation of smaller particles. This effect is attributed to pH-induced alterations in the surface charge of nanoparticles and modifications in the surrounding dielectric environment^73^.

### Effect of temperature on the biosynthesized Jan-AuNPs

Temperature significantly influenced the biosynthesis efficiency of Jan-AuNPs. Temperature plays a pivotal role in Jan-AuNPs biosynthesis and exhibits significant mutual interaction with both incubation time and gold ion concentration (Fig. 6D, E). Maximum Jan-AuNPs yield (289 µg/mL) was achieved at approximately 60 °C, with an incubation time of 3 h, and gold concentration of 300 µg/mL. Lower temperatures (< 45 °C) reduced reaction rates, while excessive heat (> 60 °C) caused thermal denaturation of biomolecules in the *J. rubens* extract. Likewise, incubation times below 1.5 h or beyond 3.5 h led to reduced Jan-AuNPs biosynthesis, possibly due to insufficient reduction or the destabilization and aggregation of Jan-AuNPs. A synergistic interaction between temperature and gold concentration was also evident. While adequate gold ion availability is crucial for Jan-AuNPs biosynthesis, the efficiency of the bio-reduction process is strongly dependent on temperature. Yields peaked at 280–300 µg/mL when synthesis was conducted at 60 °C with gold concentrations of 250–300 µg/mL. Inadequate temperatures or insufficient gold ion concentrations (< 150 µg/mL) led to reduced Jan-AuNPs yield. High gold ion concentrations at elevated temperatures may promote uncontrolled nucleation and Jan-AuNPs aggregation, disrupting the stability of Jan-AuNPs.

Temperature plays a crucial role in influencing both the efficiency and rate of AuNPs biosynthesis. Hokmollahi et al.^74^ reported that higher temperatures significantly accelerated nanoparticles’ formation, primarily by reducing the overall synthesis time. This is attributed to increased nucleation rates at elevated temperatures, which often result in the formation of smaller nanoparticles.

Similarly, Ghaemi and Gholamipour^75^ observed that higher temperatures facilitated the faster reduction of gold ions, thereby enhancing the biosynthesis process. Supporting this, Shah et al.^76^ found that rising temperatures not only increased the conversion rate of gold ions but also led to a reduction in the average nanoparticles’ size. Rai et al.^77^ demonstrated a direct correlation between temperature and reaction rate during the synthesis of triangular AuNPs using *Cymbopogon flexuosus* extract, with higher temperatures promoting faster nanoparticles’ formation. This was further supported by Pandey et al.^78^, who observed that the biosynthesis process occurred rapidly at 60, 80, and 100 °C, typically marked by an immediate color change, indicating the successful formation of gold nanoparticles. Costa et al.^79^ reported that *Sargassum cymosum* produced spherical AuNPs ranging from 5 to 22 nm under optimal conditions between 60 and 80°C. Likewise, Rajeshkumar et al.^80^ identified 80°C as the optimal temperature for the biosynthesis of spherical AuNPs (8–10 nm) using *Padina tetrastromatica* extract.

### Effect of incubation time on the biosynthesized Jan-AuNPs

The incubation time is a crucial parameter in Jan-AuNPs biosynthesis, especially in synergy with gold concentration (Fig. 6F). The interaction between gold ion concentration and incubation time significantly influenced the biosynthesis efficiency of Jan-AuNPs. Optimal biosynthesis (289 µg/mL) was achieved at 3–3.5 h incubation and > 250 µg/mL gold concentration, likely due to the sufficient time allowed for complete reduction and stabilization of Jan-AuNPs by active biomolecules in the *J. rubens* extract. These findings suggest that prolonged exposure allows the biomolecules in *J. rubens* sufficient time to reduce Au³⁺ ions and stabilize the resulting Jan-AuNPs. Extended incubation beyond optimal times did not further enhance yield and may promote agglomeration or cause capping agent degradation or destabilization. Longer incubation times (3–3.5 h), when combined with high gold ion concentrations (> 250 µg/mL), produced the highest yield of Jan-AuNPs. This enhancement can be attributed to two main factors: (i) adequate time for biomolecules to complete the reduction of Au³⁺ ions and effectively stabilize the resulting nanoparticles, and (ii) the presence of sufficient gold ions to support maximum Jan-AuNPs nucleation. However, both excessive gold concentration and prolonged incubation time may lead to Jan-AuNPs agglomeration, particularly if sufficient capping agents are not present to prevent instability.

Singaravelu et al.^81^ demonstrated that the extracellular biosynthesis of AuNPs using *Sargassum wightii* occurred rapidly, with nanoparticles’ formation taking place within a brief incubation period. This supports current observations and suggests that the biomolecules responsible for reducing gold ions are highly active during the early stages of the reaction. In contrast, Isaac and Renitta^82^ reported that *Padina pavonica* required a longer incubation period of 24 h to produce AuNPs, which were predominantly spherical and ranged in size from 35.4 to 69.6 nm, even at low gold ion concentrations. The extended synthesis time in that case may be attributed to differences in algal composition, extract preparation methods, or reaction conditions. Similarly, *Galaxaura elongata* was capable of synthesizing rod-shaped and triangular AuNPs over a broad incubation range of 10 min to 12 h under continuous stirring at 120 rpm^83^. *Lemanea fluviatilis* required 12 h of incubation at room temperature with stirring to produce spherical nanoparticles ranging from 5 to 15 nm in size^84^. Mata et al.^70^ observed that *Fucus vesiculosus* synthesized spherical AuNPs (20–50 nm) after 1–8 h of incubation across a wide pH range (2–9). In another study, *Turbinaria conoides* produced polydispersed AuNPs of various shapes, including rectangular and triangular forms, after 24 h of incubation^85^.

### Effect of gold ion concentrations on the biosynthesized Jan-AuNPs

Gold ion concentration significantly influenced the biosynthesis yield of Jan-AuNPs. As the concentration of gold ions increased, Jan-AuNPs production improved, reaching a peak at concentrations above 250 µg/mL (Fig. 6C, E, F). However, beyond this optimal level, the yield began to plateau or decline, likely due to oversaturation of the reaction medium, which may induce uncontrolled nucleation and aggregation. The initial increase is attributed to the greater availability of Au³⁺ ions, which promotes efficient nucleation and subsequent nanoparticles’ growth^86^. However, at higher concentrations, the reaction system may become oversaturated, leading to uncontrolled nucleation and increased risk of particles’ agglomeration. Therefore, maintaining an optimal gold ion concentration is crucial for producing Jan-AuNPs with high yield, stability, and uniform size. In the case of *Padina tetrastromatica*, no SPR peak was observed at a gold ion concentration of 0.1 mM. However, at 0.5 mM, a broad SPR band emerged around 536 nm. Increasing the concentration to 1 mM resulted in a shift of the SPR peak to 532 nm, indicating the formation of smaller and more homogeneous AuNPs. Further elevation of the gold concentration to 2 mM caused a shift to 541 nm, suggesting the synthesis of larger nanoparticles. Based on these observations, 1 mM was identified as the optimal concentration for producing well-defined AuNPs using *P. tetrastromatica* extract^87^. Similarly, Singh et al.^88^ reported that stable AuNPs ranging from 8 to 21 nm in size were synthesized using 1 mM HAuCl₄ and 5% algal biomass of *Padina gymnospora* at 75 °C. Variations in the gold concentration were shown to influence nanoparticles’ morphology, leading to the generation of diverse structures with potential applications in semiconductors and drug delivery systems^89^.

### Evaluation of the model’s adequacy

The normal probability plot (NPP) of the residuals is a crucial graphical tool used to assess the adequacy of a statistical model^90^. It visually displays the distribution of residuals to determine whether they follow a normal distribution to confirm the adequacy of the model^91^.

**Fig. 7:**
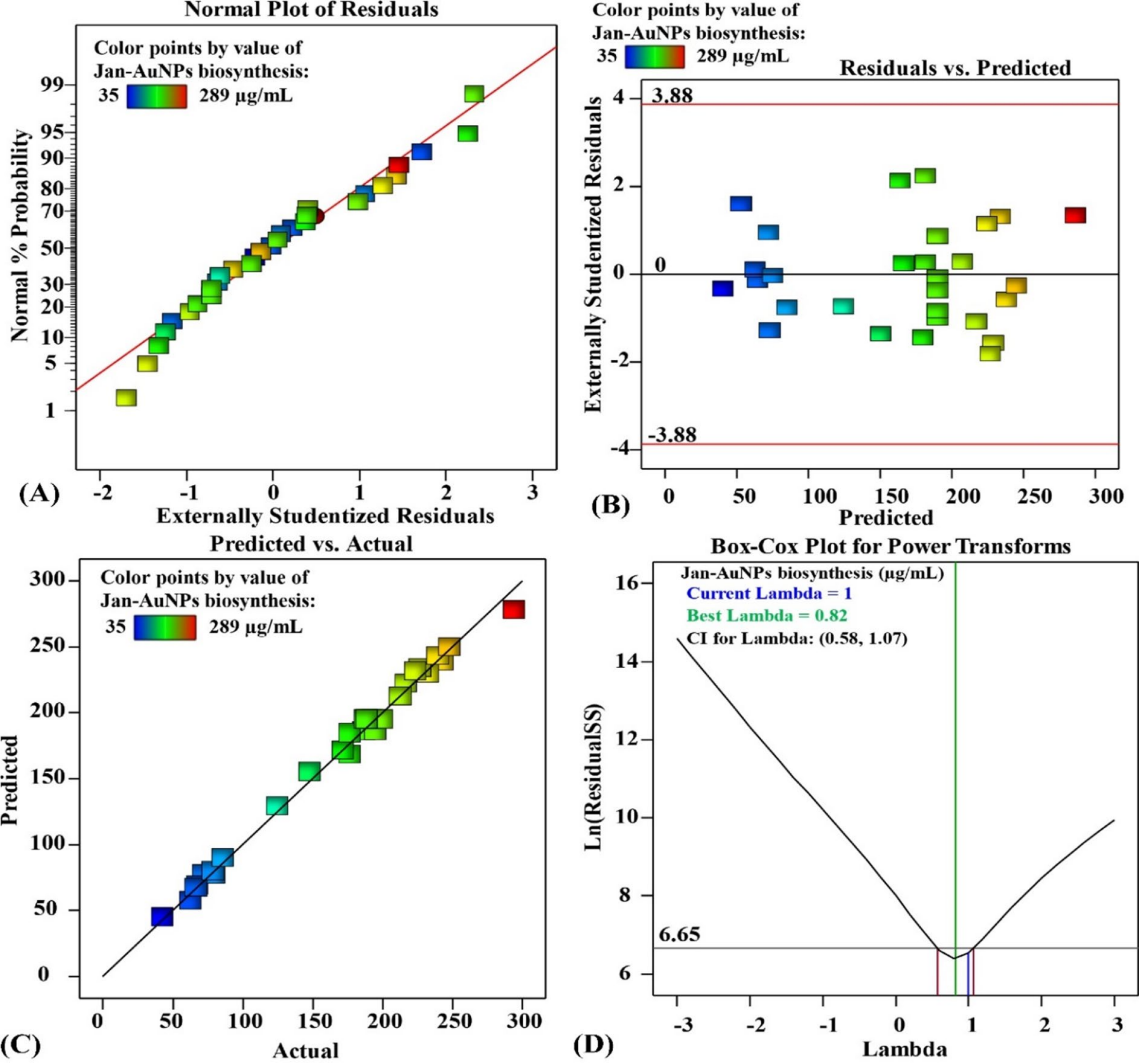
(**A**) Normal plot of residuals, (**B**) plot of residuals versus predicted values, (**C**) plot of actual versus predicted values, and (D) Box-Cox plot for power transforms of Jan-AuNPs biosynthesis by *J. rubens* as affected by initial pH level, temperature, incubation period, and gold concentration.

Figure 7 A illustrates that the residuals are closely distributed along the diagonal line of the normal probability plot, indicating that the residuals are normally distributed. This pattern confirms the adequacy of the model used for predicting the biosynthesis of Jan-AuNPs. Residuals represent the differences between the observed (actual) and predicted values, and their distribution provides insight into model performance. The small and randomly scattered residuals observed suggest minimal variation between predicted and actual values, further supporting the accuracy and reliability of the model.

Figure 7B presents the studentized residuals plotted against the predicted values of Jan-AuNPs biosynthesis. The residuals appear randomly scattered around the zero line, indicating that the variance of errors remains consistent across the range of predicted values and confirming the adequacy of the regression model. Additionally, the residuals are symmetrically distributed above and below the horizontal axis, further supporting the presence of constant error variance across the predicted values. Moreover, all data points fall within the control limits of ± 3, suggesting that none of the studentized residuals exceed the acceptable range and confirming the absence of extreme data points that deviate significantly from the overall pattern of the dataset that could affect the accuracy and reliability of the statistical model.

Figure 7C illustrates the correlation between the predicted and actual values of Jan-AuNPs biosynthesis, serving as a key indicator of the model’s predictive accuracy. The data points are closely aligned along the reference line, demonstrating a strong agreement between the experimental and predicted responses^92^. This close alignment reflects the high precision and reliability of the regression model in reflecting the relationship between the experimental variables and the Jan-AuNPs synthesis.

In Fig. 7D, the blue line represents the current Lambda (λ = 1) and the green line represents the best Lambda value (λ = 0.82). Whereas the two red lines represent the minimum and maximum 95% confidence interval values (0.58 and 1.07, respectively). The model requires no transformation since it is in the optimal zone, because the current Lambda’s blue line falls between the two red lines. As a result, the model is well-fitted to the experimental results and data.

### Desirability function (DF)

**Fig. 8:**
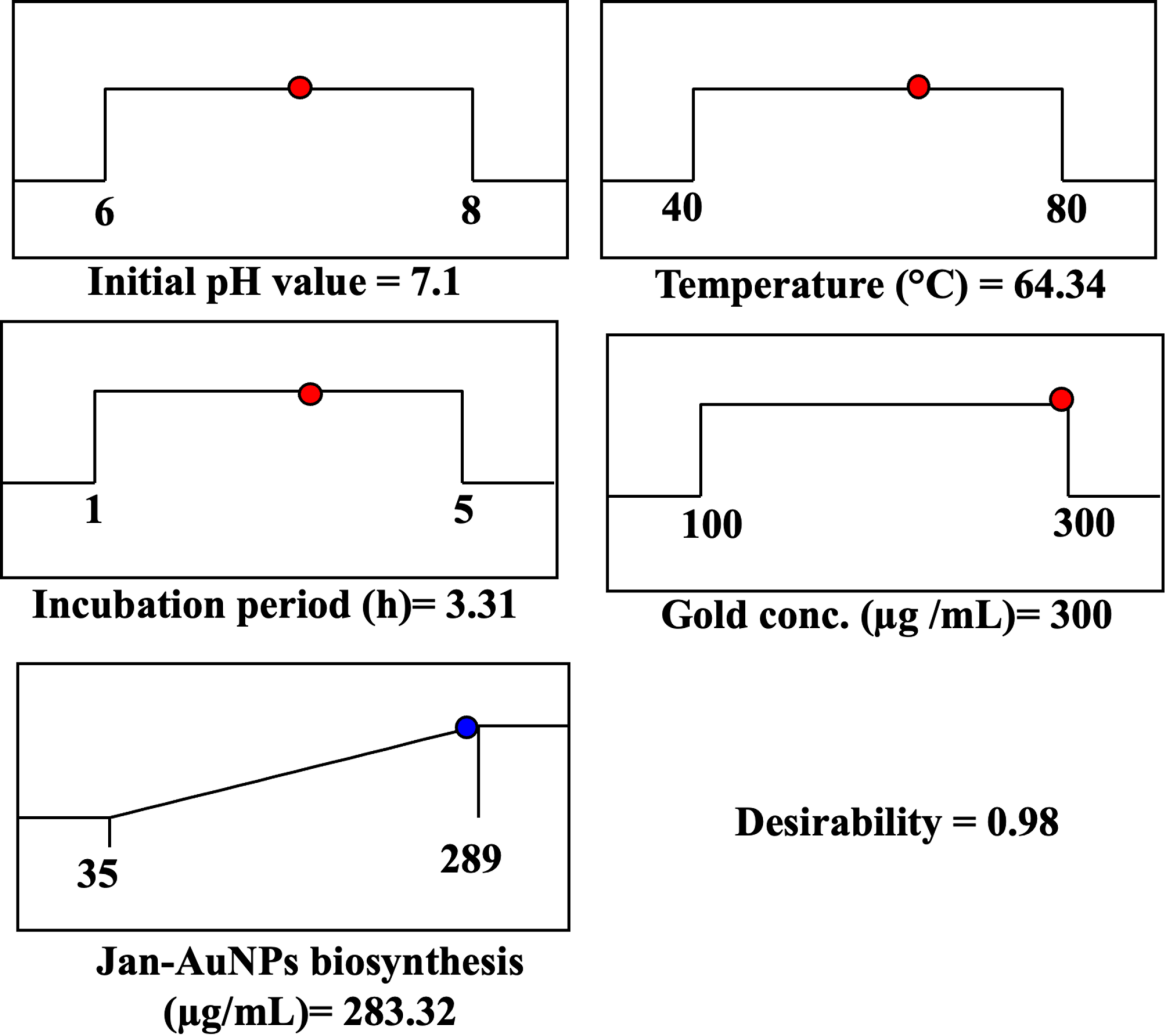
Desirability function plot reveals the optimum predicted values for maximum Jan-AuNPs biosynthesis by *J. rubens* and the desirability value.

The desirability function (DF) is a widely utilized technique for optimizing multivariate systems, enabling the identification of the optimal predicted conditions that maximize the response^93^. Using the DF approach within Design Expert 12.0 software, the optimization plot (Fig. 8) reveals the predicted conditions for maximizing Jan-AuNPs biosynthesis. The model estimates a maximum yield of 283.32 µg/mL at an initial pH of 7.1, temperature of 64.34 °C, incubation time of 3.31 h, and a gold ion concentration of 300 µg/mL with a high desirability value of 0.98. Experimental validation using the optimized parameters yielded a Jan-AuNPs production of 295 µg/mL, demonstrating close alignment with the model’s predicted conditions and confirming the robustness and predictive accuracy of the optimization process.

### *In vitro* cytotoxicity of Jan-AuNPs against cancer and normal cells

**Fig. 9:**
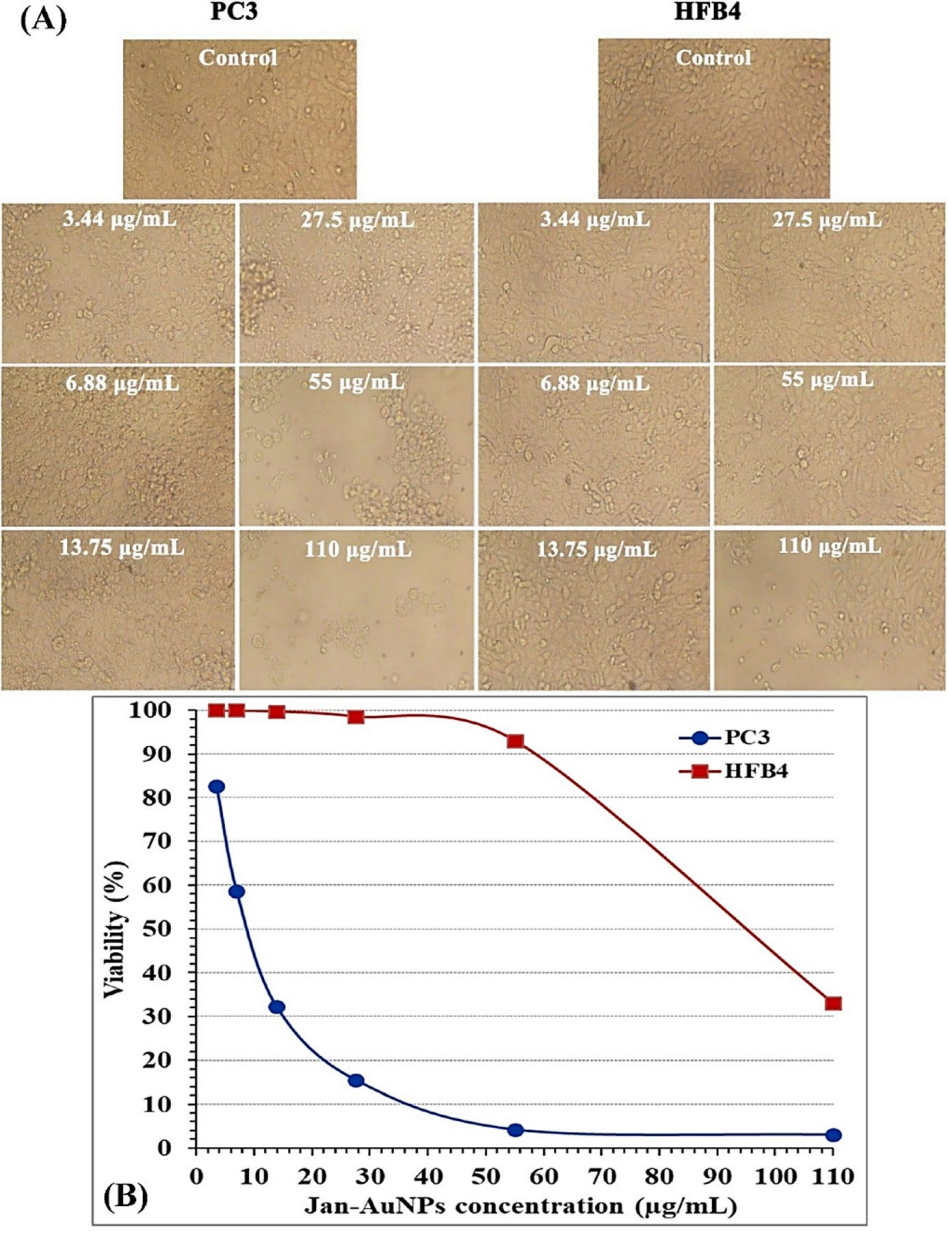
(**A**) *In vitro* morphological observation by inverted microscope (10X) of human cancer prostate cells (PC3) and normal skin cells (HFB4) treated with the biosynthesized Jan-AuNPs at different concentrations. Scale bar represents 100 μm. (**B**) Cell viability (%) was calculated relative to the untreated control (100%). The results show dose-dependent cytotoxicity of Jan-AuNPs. Data are presented as mean ± SD (*n* = 3).

Figure 9 illustrates the *in vitro* anticancer activity of biosynthesized Jan-AuNPs against human prostate cancer cells (PC3) and normal human skin fibroblasts (HFB4). The cytotoxicity was assessed by determining the half-maximal inhibitory concentration (IC₅₀) values after 48 h of treatment, relative to untreated controls. Jan-AuNPs demonstrated potent cytotoxic effects against PC3 cells, with an IC_50_ value of 6.39 µg/mL, whereas a higher IC_50_ value of 97.41 µg/mL was observed for HFB4 cells. One-way ANOVA and Tukey’s post hoc test were used to statistically examine the cytotoxic effect of Jan-AuNPs on HFB4. The tested concentrations showed a highly significant difference (*F* = 1520.7, *P* < 0.0001). The results of post hoc analysis showed that Jan-AuNPs at 110 µg/mL and 55 µg/mL significantly decreased cell viability when compared to the control group (*P* < 0.001 and *P* = 0.012, respectively), while no significant changes were observed at the other concentrations (*P* > 0.05). For PC3, the tested concentrations showed a highly significant difference (*F* = 1987.4, *P* < 0.0001). All Jan-AuNPs concentrations resulted in a significant decrease in cell viability when compared to the control group, according to post hoc analysis (*P* < 0.01). This pronounced difference indicates selective toxicity towards the cancerous cell line. To quantify this selectivity, the selectivity index (SI) was calculated using the formula: SI=IC_50_ in a normal cell line/IC_50_ of Jan -AuNPs in the cancer cell line, where IC_50_ is the concentration required to kill 50% of the cell population^94^. The resulting SI value for Jan-AuNPs reached 15.24. This value means that Jan-AuNPs were approximately 15 times more toxic to cancer cells than to normal cells. According to Bézivin et al.^95^, an SI greater than 3 is considered highly promising, while Suffness and Pezzuto^96^ suggest an SI of 2 or more indicates a compound of interest for anticancer development. These findings highlight the selective cytotoxicity of Jan-AuNPs toward prostate cancer cells, along with their minimal toxicity on normal cells, supporting their continued advancement as innovative candidates in the field of cancer nanotherapy.

Although the cytotoxicity of the *J. rubens* extract alone was not determined, the biosynthesized AuNPs were subjected to repeated washing and centrifugation to eliminate any unbound algal residues. Therefore, the pronounced cytotoxic effect against PC3 cells is primarily attributed to the nanoparticles themselves. However, the surface-bound biomolecules derived from the *J. rubens* extract act as capping and stabilizing agents, forming an organic layer around the nanoparticles that contributes to their stability and surface functionality. These surface-bound compounds may contribute to the biological behavior by enhancing cellular uptake or promoting specific molecular interactions at the cell membrane. Such synergistic effects between the gold core and bio-capping layer could partially explain the enhanced cytotoxic efficiency observed for the biosynthesized AuNPs compared with other reported systems.

Numerous studies have investigated the cytotoxic effects of green-synthesized and functionalized AuNPs on the PC3 prostate cancer cell line, reporting a wide range of IC_50_ values influenced by factors such as the biological source of the extract, synthesis technique, and physicochemical properties of the nanoparticles. For instance, Vinay et al.^97^ reported an IC_50_ value of 64.23 µg/mL for AuNPs synthesized using *Elaeocarpus ganitrus* seed extract. In contrast, Prema et al.^98^ observed enhanced cytotoxicity with AuNPs derived from *Camellia sinensis* L., which exhibited an IC_50_ of 19.71 µg/mL against PC3 cells. Similarly, Al-Abdeli and Al-kalifawi^99^ demonstrated that AuNPs biosynthesized using *Acinetobacter baumannii* induced significant apoptosis, with an IC_50_ of 13.72 µg/mL. A time-dependent cytotoxicity trend was reported by Yayintas et al.^100^, who observed IC_50_ values of 39.84 µg/mL after 24 h and 25.05 µg/mL after 48 h for AuNPs biosynthesized by *Nasturtium officinale* extract. The underlying mechanism of AuNPs-induced cytotoxicity has been linked to their cellular uptake and the subsequent generation of reactive oxygen species (ROS). According to Rahman et al.^101^, nanoparticles are absorbed by cancer cells, trapping free electrons and inducing the generation and accumulation of intracellular reactive oxygen species (ROS). These ROS then interact with cellular proteins to cause oxidative stress.

### *In vivo* cytotoxicity of Jan-AuNPs

**Fig. 10:**
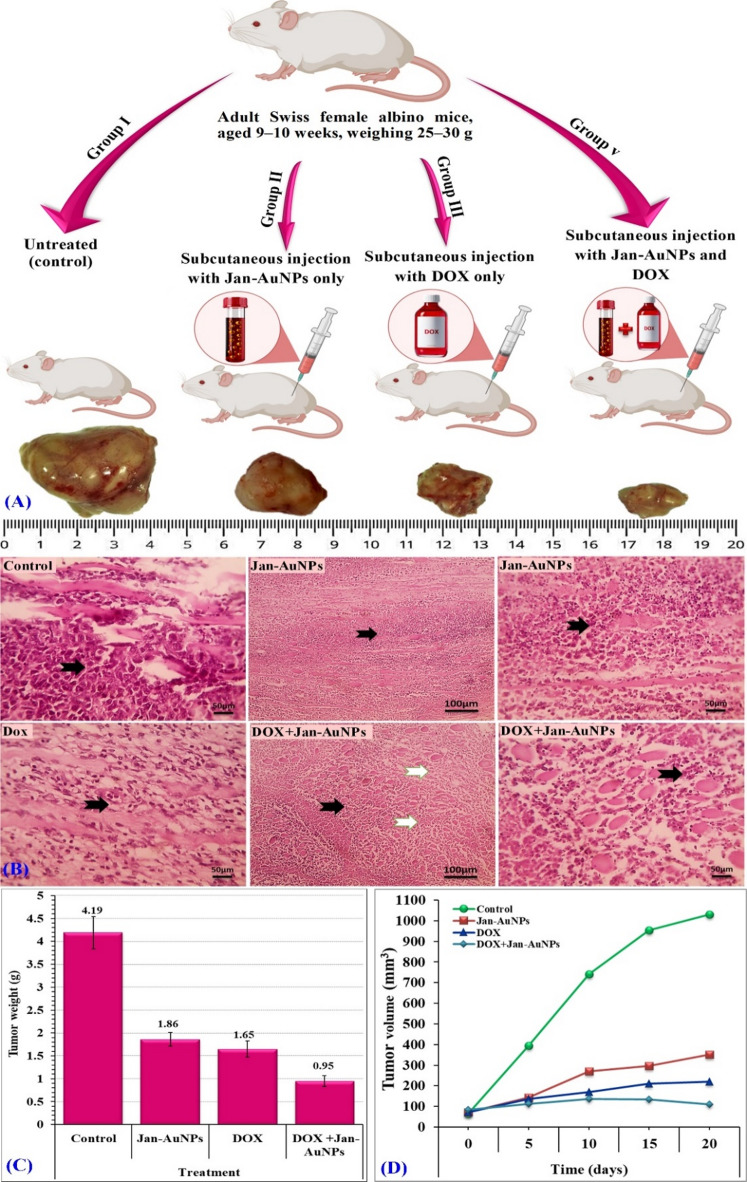
*In vivo* cytotoxicity of Jan-AuNPs, DOX, and Jan-AuNPs + DOX on EST-bearing mice with images of solid tumors at the same power of magnification, zooming, and distance from the camera, histopathological analysis, and their effects on tumor weight and volume.

The present study investigated the antitumor efficacy of biosynthesized Jan-AuNPs, alone and in combination with Doxorubicin (DOX), against Ehrlich ascites carcinoma (EAC) in Swiss albino mice. Four experimental groups were established to evaluate the effects of each treatment: untreated control, Jan-AuNPs, DOX, and a combination of DOX and Jan-AuNPs (Fig. 10; Table 6). Visual inspection of excised tumors clearly demonstrated a substantial reduction in tumor mass among treated groups compared to the control. The untreated group (Group I) exhibited large, highly vascularized tumors indicative of aggressive tumor proliferation (tumor volume increased from 0.06 ± 0.01 to 1.03 ± 0.07 cm^3^). The untreated group displayed the highest average tumor weight (4.19 g), reflecting unchecked tumor progression. In contrast, mice treated with Jan-AuNPs alone (Group II) displayed noticeably smaller tumors. Jan-AuNPs alone significantly reduced tumor weight to 1.86 g, suggesting effective suppression of tumor growth. The tumor volume was reduced to 0.35 ± 0.08 cm³, and its weight decreased to 1.86 ± 0.15 g. This reduction corresponded to a ΔT/ΔC value of 28.86%, indicating a 71.14% inhibition in tumor growth. Furthermore, the MST was prolonged to 35 days, suggesting a modest but significant improvement in survival and therapeutic effect. DOX monotherapy yielded a slightly greater reduction to 1.65 g, consistent with its established chemotherapeutic action.

**Table 6:** Inhibition effect of Jan-AuNPs, DOX, and DOX + Jan-AuNPs on EAT-bearing mice tumor.

Groups	Tumor weight (g)	Tumor dimensions (cm)				Tumor volume (cm^3^)		ΔT/ΔC (%)	Inhibition (%)	MST (day)
		0 day		20 day		0 day	20 day			
		S	L	S	L					
Control	4.19 ± 0.35	0.45 ± 0.01	0.63 ± 0.04	1.09 ± 0.03	1.74 ± 0.05	0.06 ± 0.01	1.03 ± 0.07	100.00	0.00	23
Jan-AuNPs	1.86 ± 0.15	0.46 ± 0.04	0.65 ± 0.05	0.76 ± 0.06	1.21 ± 0.09	0.07 ± 0.01	0.35 ± 0.08	28.86	71.14	35
DOX	1.65 ± 0.18	0.47 ± 0.02	0.63 ± 0.06	0.65 ± 0.02	1.04 ± 0.5	0.07 ± 0.01	0.22 ± 0.02	15.46	84.54	44
DOX + Jan-AuNPs	0.95 ± 0.12	0.49 ± 0.03	0.68 ± 0.04	0.55 ± 0.07	0.72 ± 0.06	0.08 ± 0.01	0.11 ± 0.02	3.09	96.91	63

ΔT and ΔC are the change of tumor volume in the treated and control groups, respectively; mean survival time (MST).

Notably, the DOX + Jan-AuNPs group (Group IV) showed the smallest tumors, strongly suggesting a synergistic effect between Jan-AuNPs and DOX. The final tumor weight and volume were significantly reduced to 0.95 ± 0.12 g and 109.36 mm³, respectively, indicating a synergistic anticancer effect. This corresponds to a tumor growth inhibition rate of 96.91%, with only 3.09% residual tumor growth relative to the control group. This reduction may be due to the enhanced cellular penetration and localized action of DOX facilitated by Jan-AuNPs, which can act as nanocarriers or therapeutic agents themselves. Additionally, the mean survival time for this treatment group reached 63 days, which was nearly three times longer than that of the untreated controls, demonstrating a substantial therapeutic advantage over monotherapy with either DOX or Jan-AuNPs alone. El-Naggar et al.^7^ reported that treatment with AuNPs resulted in a 71.6% tumor growth inhibition in EST-bearing mice. Notably, the combination of AuNPs with Doxorubicin (DOX) enhanced this effect, achieving a tumor growth inhibition rate of 90.5%.

Similarly, in a study involving EAC-bearing mice, myco-synthesized AuNPs produced using an aqueous extract of *Cladosporium* sp. exhibited potent cytotoxic activity. This treatment significantly reduced body weight and ascites volume while also prolonging the survival of the treated animals^102^. The observed synergy may be attributed to the anticancer properties of Jan-AuNPs, which include the induction of oxidative stress, disruption of mitochondrial function, and DNA fragmentation. The enhancement of DOX activity when co-administered with Jan-AuNPs might also be attributed to improved cellular uptake or increased intracellular retention of DOX facilitated by nanoparticles-mediated delivery, thereby potentiating its cytotoxic effects and promoting more effective tumor cell targeting. Histological analysis of tumor sections stained with hematoxylin and eosin (Fig. 10) showed different histopathological characteristics between the treatment groups. The control group exhibited dense, numerous large malignant cells exhibiting anaplasia, pleomorphism, nuclear dyschromasia, and atypical nuclei, consistent with aggressive malignancy.

Jan-AuNPs-treated tumors revealed improved cell structure and reduced tumor growth, necrotic areas (without eosinophilic structures), more apoptotic bodies, and a slight improvement in histology. DOX-treated tumors showed partial tumor regression, characterized by the presence of apoptotic bodies, nuclear condensation, and some tissue reorganization. It is noteworthy that the group treated with a mixture of DOX and Jan-AuNPs showed pronounced nuclear sclerosis and clear signs of restoration of normal cell structure. These findings confirm a synergistic interaction between Jan-AuNPs and DOX, leading to superior therapeutic outcomes compared to monotherapy with either DOX or Jan-AuNPs alone. This synergistic interaction is reflected in the pronounced tumor volume and weight reduction, along with a substantial increase in survival time. Overall, Jan-AuNPs demonstrate strong potential as a synergistic nanocarrier in cancer therapy, enhancing drug effectiveness and lowering systemic toxicity. As summarized in Table 7, previous studies have reported various approaches employing AuNPs alone or in combination with chemotherapeutic agents to enhance antitumor activity against Ehrlich carcinoma.

Extensive research has established a strong correlation between the size of AuNPs and their cellular uptake. Ultra-small AuNPs exhibit superior internalization, enhanced nuclear penetration in cancer cells, and reduced cytotoxicity compared to their larger counterparts^122^. Jan-AuNPs exert anticancer effects through multiple complementary mechanisms in both EAC-bearing animal models and prostate cancer cell lines. Owing to the enhanced permeability and retention (EPR) effect, Jan-AuNPs preferentially accumulate within tumor tissues following administration, resulting in higher localization within malignant cells compared to normal tissues^123^.

**Table 7:** Comparative summary of previous studies evaluating the antitumor efficacy of biosynthesized AuNPs.

Type / source of AuNPs	Treatment groups	Tumor growth inhibition / cytotoxicity	Main findings	References
Jan-AuNPs (biosynthesized using *Jania rubens*)	Jan-AuNPs; DOX + Jan-AuNPs	71.14%; 96.91%	Synergistic anticancer effect; enhanced DOX uptake; prolonged survival and improved histopathology.	This study
AuNPs synthesized using*Streptomyces flavolimosus*)	AuNPs; AuNPs + DOX	AuNPs (71.6%); AuNPs + DOX (90.5%)	Combination therapy enhanced DOX efficacy; reduced tumor size and systemic toxicity.	El-Naggar et al.^7^
Myco-synthesized AuNPs (*Cladosporium* sp.)	AuNPs only		Decreased ascitic volume and tumor cell count; prolonged survival.	Munawer et al.^102^
EGCG-capped AuNPs (Epigallocatechin-3-gallate)	EGCG–AuNPs		Selective cytotoxicity to tumor cells; reduced tumor size and minimal toxicity.	Safwat et al.^103^
PST–AuNPs (Galactoxyloglucan polysaccharides)	PST–AuNPs		Reduced tumor weight and volume; enhanced apoptosis.	Joseph et al.^104^
Dextran-coated AuNPs (*leuconostoc* spp.)	Au–Dextran NPs		Significant inhibition of tumor growth; improvement in biochemical parameters.	Medhat et al.^105^
Thymoquinone-loaded AuNPs	AuNPs–TQ		Enhanced apoptosis; increased antioxidant activity; potent tumor inhibition.	Gomaa et al.^106^
AuNPs synthesized using *Nasturtium officinale* L.	NO-AuNPs vs. A549 cells	Significant reduction in lung cancer cell viability	Activation of apoptosis and cell cycle arrest	Yayintas et al.^107^
AuNPs synthesized using *Acinetobacter baumannii*	AuNPs	IC_50=_ 11.45 µg/mL (cytotoxic)	Strong *in vitro* anticancer against breast cancer cells	Al-Abdeli & Al-Kalifawi^108^
AuNPs synthesized using *Scabiosa palaestina* extract	AuNPs	Dose-dependent inhibition (various IC_50_)	Potent antiproliferative activity on MDA-MB-231, HCT116 & 22Rv1	Hellany et al.^109^
Plant-mediated AuNPs (*Cirsium japonicum var. maackii*)	CJ-AuNPs vs. control & 5-FU in xenograft mice	62.5–71.9% reduction in tumor volume/weight	Inhibits tumor growth in gastric cancer xenograft model	Mi et al. ^110^
Biosynthesized AuNPs (Peltophorum pterocarpum leaves)	PSP-AuNPs vs. control in tumor-bearing mice	Significant inhibition of tumor growth vs. DOX alone (*in vivo*)	Enhanced anticancer efficacy of doxorubicin	Mukherjee et al. ^111^
AuNPs synthesized using leaf extracts (*Carica papaya* & *Catharanthus roseus*)	AuNPs vs. HepG2 & MCF-7	Significant cytotoxicity (*in vitro*)	Green AuNPs showed enhanced anticancer activity	Muthukumar et al. ^112^
AuNPs synthesized using *Taxus baccata* ethanolic extract	AuNPs vs. MCF-7, HeLa, Caov-4	High cytotoxicity on multiple cancer lines	Induced cancer cell death via caspase-independent mechanisms	Kajani et al. ^113^
AuNPs from *Commiphora wightii*	AuNPs vs. MCF-7	Induced G2/M cell cycle arrest	Plant-mediated anticancer effect	Uzma et al. ^114^
AuNPs synthesized using *Punica granatum* extract	AuNPs vs. MCF-7	Caused apoptosis via G0/G1 arrest	Promoted DNA fragmentation	Ganeshkumar et al. ^115^
AuNPs synthesized using *Turnera diffusa* extract	AuNPs vs. HL-60	Strong cytotoxic effects on leukemia cells	*In vitro* anticancer potential	Reyes-Becerril et al.^116^
AuNPs synthesized using *Mentha longifolia*	AuNPs vs. various breast cancer lines	Significant dose-response cytotoxicity	Indicated anticancer activity	Li et al. ^117^
AuNPs synthesized using *Hibiscus rosasinensis*	AuNPs vs. breast cancer cells	Strong antitumor effects *in vitro*	Demonstrated high cytotoxicity levels	Yasmin et al. ^118^
AuNPs synthesized using *Boerhavia diffusa* (BD) extract	BD-AuNPs	39% viability decrease in HepG2 cells	Anticancer activity against liver cancer line	Bibi et al. ^119^
AuNPs synthesized using *Tecoma capensis* leaf extract	AuNPs vs. cancer cells	Dose-dependent cytotoxicity	Strong anticancer & antioxidant responses	Hosny et al. ^120^
AuNPs synthesized using *Ziziphus spina-christi* leaves	AuNPs vs. MCF-7	IC_50_ 48 µg/mL	Anticancer activity *in vitro*	Hosny et al. ^121^

Upon internalization by cancer cells, Jan-AuNPs can induce excessive generation of reactive oxygen species (ROS), surpassing the cellular antioxidant defense capacity and causing oxidative damage to proteins, lipids, and DNA. This oxidative stress can lead to mitochondrial dysfunction, leading to activation of the intrinsic apoptotic pathway characterized by cytochrome-c release and sequential activation of caspase-9 and caspase-3, ultimately resulting in apoptotic cell death^124^. In prostate cancer cell lines, AuNPs have been reported to induce DNA fragmentation and morphological alterations characteristic of apoptosis, accompanied by a significant increase biochemical markers of apoptosis compared to control treatments^125^. AuNPs have been demonstrated to inhibit tumor growth, decrease tumor weight and volume, and decrease the number of viable tumor cells in the EAC model *in vivo*. These antitumor effects are accompanied by apoptotic induction and tumor microenvironment modification, which improves the therapeutic effect without causing significant toxicity to healthy tissues^8^. Accordingly, Jan-AuNPs exert their anticancer activity through multiple interconnected mechanisms, including ROS generation, DNA damage, activation of the intrinsic apoptotic pathway, and selective accumulation within tumor tissues.

The liver and kidneys represent the primary organs responsible for the clearance of nanomaterials from the body, and the specific elimination pathway largely depends on the size of the NPs. Small AuNPs are mainly excreted from the body through the renal route, as their relatively small size enables them to pass through the kidneys and thereby avoid prolonged retention within biological tissues^126^. The renal clearance process typically involves the filtration of smaller nanoparticles from the bloodstream, leading to their elimination within hours to a few days following administration. When the diameter of AuNPs exceeds the renal filtration cutoff, their removal shifts to the reticuloendothelial system, where these particles are captured and eliminated, primarily within the liver. Hepatobiliary excretion serves as the predominant pathway, whereby AuNPs are secreted from the liver into the bile ducts, transported to the intestines, and ultimately expelled via the feces over a period ranging from several hours to weeks post-administration. The reticuloendothelial system also plays a key role in the long-term retention of non-biodegradable nanoparticles, sometimes trapping them for periods exceeding six months. Numerous studies have confirmed that AuNPs are cleared through these pathways depending on factors like particle size, shape, and surface properties. Consequently, the kidneys, liver, and spleen are regarded as the principal organs investigated when assessing the biodistribution, metabolism, and clearance of AuNPs^127,128^.

### *In silico* computational analysis

**Fig. 11:**
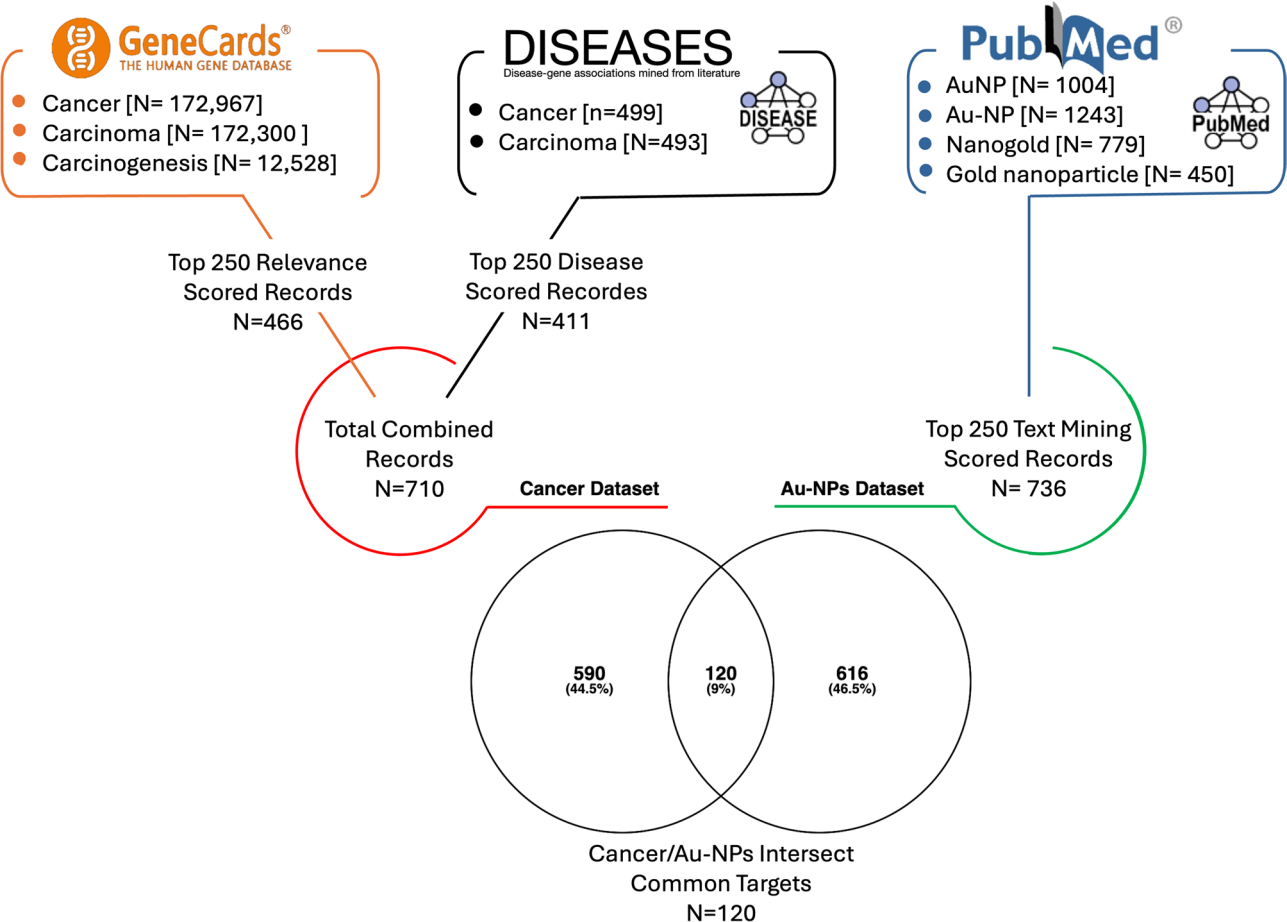
Cancer/AuNPs target prediction workflow. A Venn diagram represents the intersecting common targets (*n* = 120).

**Fig. 12:**
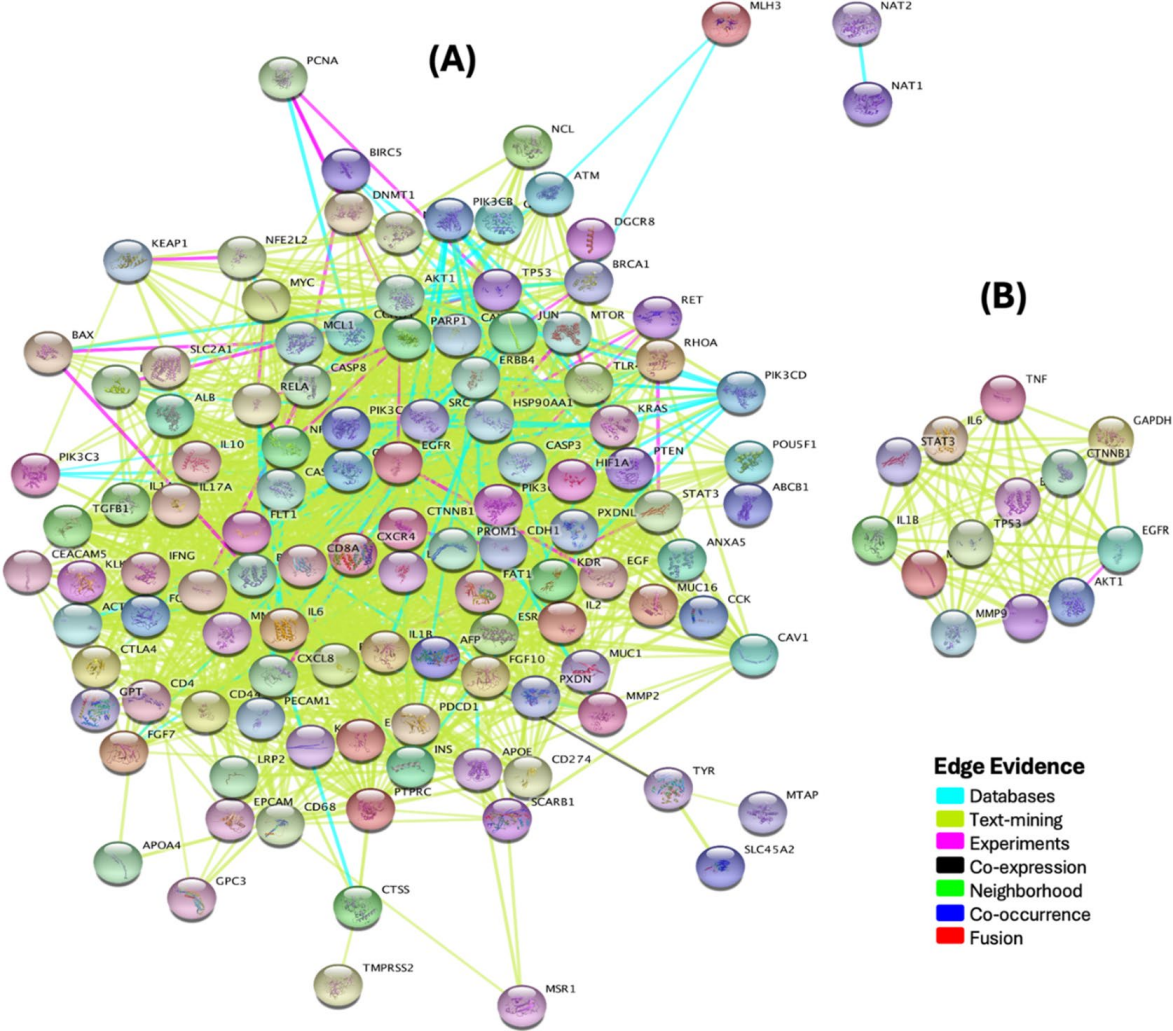
Protein-protein interaction (PPI) network of (**A**) Cancer/AuNPs target genes (degree ≥ 24.4) and (**B**) gene hubs (degree ≥ 50).

The current *in silico* computational analysis, including protein-protein interaction (PPI) network construction, gene ontology (GO), KEGG pathway and metabolic module enrichment analyses, aims to predict the cancer type most likely affected by AuNPs based on identifying cancer-associated gene targets modulated by AuNPs. This analysis utilizes datasets curated from multiple human disease/gene databases (Figs. 11, 12 and 16A,B; Table 8), to reveal the cancer type with the highest therapeutic potential of AuNPs (Fig. 16A). This approach highlights the crucial role of computational predictive models in guiding experimental research and optimizing nanoparticle-based cancer therapies for precision oncology.

### Cancer/AuNPs-related target prediction

The relevance score, disease score, and text mining score were used to filter the datasets’ gene records retrieved from GeneCards, Disease, and PubMed databases, respectively. The top 250 highly scored records of each keyword dataset were combined after removing duplicates, irrelevant, or empty records to get a single cancer dataset with 710 total records versus a single AuNPs dataset with 736 total records. The Intersect common gene records [*n* = 120] between both cancer and AuNPs datasets represent the AuNPs’ related targets with potential anticancer activity (Fig. 11).

### Protein–protein interaction (PPI) network construction and analysis

As shown in Fig. 12A, after excluding the disconnected nodes, the STRING PPI network comprised 114 nodes of the common targets and 1461 edges (average node degree of 24.4). Based on a degree centrality ≥ 50, 13 top hubs (Fig. 12B) of highly connected nodes included ACTB, AKT1, BCL2, CTNNB1, EGFR, GAPDH, IL1B, IL6, MMP9, MYC, STAT3, TNF, and TP53. These hubs are supposed to be critical to the network’s structure and function and align with their roles in cancer progression and nanoparticle-mediated targeting.

### GO, KEGG pathways, and modules enrichment analysis

**Fig. 13:**
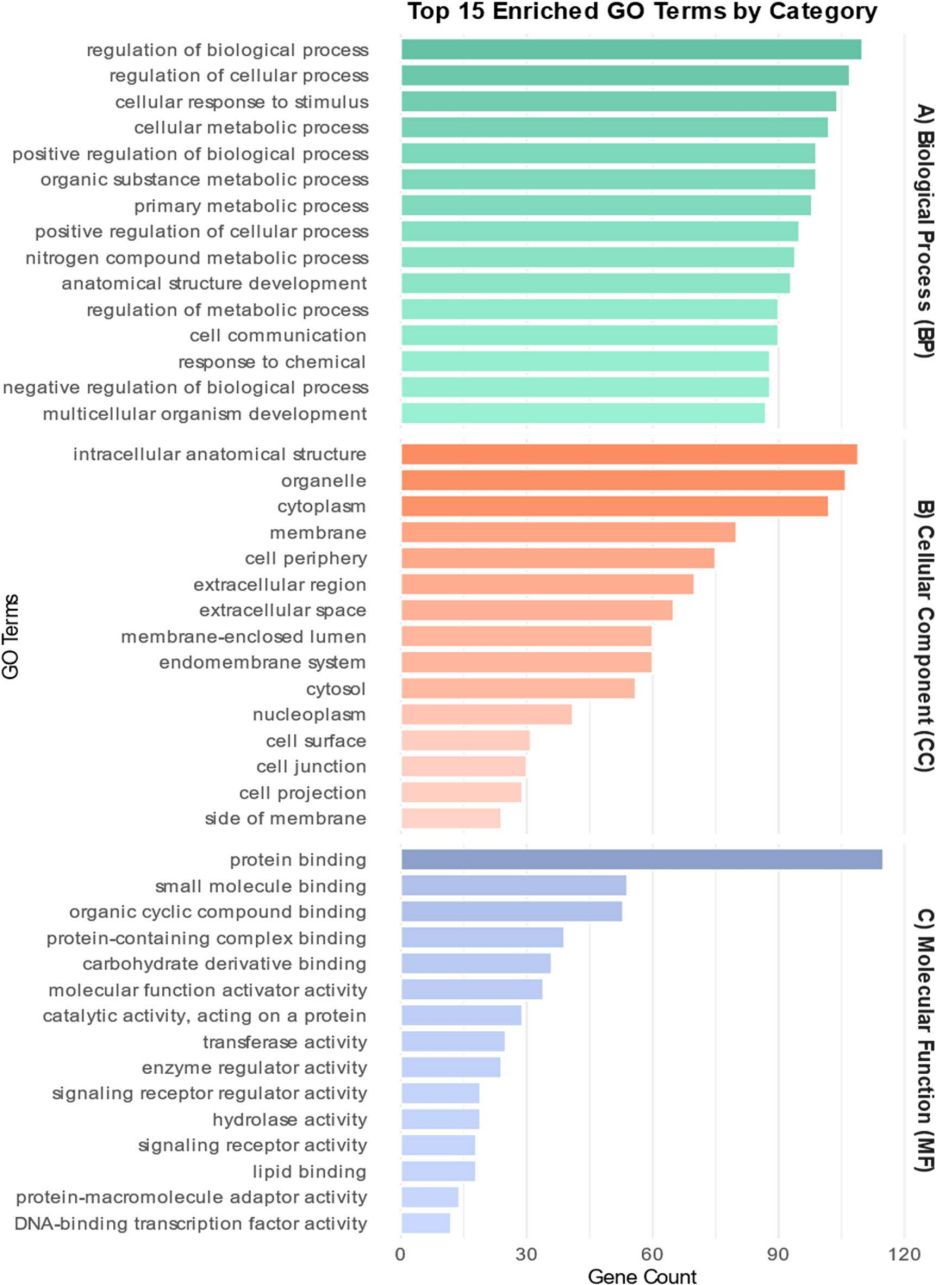
Gene ontology (GO) functional analysis of Cancer/AuNPs’ common targets. GO terms associated with biological process (BP, Green), cellular component (CC, Orange), and molecular functions (MF, blue) are expressed on the vertical y-axis, while their gene counts are expressed on the horizontal x-axis.

Biological functions and metabolic pathways of cancer/AuNPs common targets [*n* = 120] were analyzed using the *clusterProfiler* R package. The enriched analysis resulted in 230 biological processes (BP), 97 cellular components (CC), 52 Molecular functions (MF), and 169 pathways, of which the top 15 significant terms are listed in Table 8. The highest gene ratios of the common targets’ biological processes (BP) GO terms included cellular response to stimuli (GO:0051716; 86.67%) and chemicals (GO:0042221; 73.33%) as well as the regulation of biological (GO:0050789; 91.67%), cellular (GO:0050794; 89.17%) and metabolic (GO:0044237; 85%) processes (Table 8; Fig. 13A). These predominant BP GO terms, including regulation of biological and cellular processes, cellular response to stimuli, and metabolic processes, indicate the AuNPs’ potential to modulate key cellular activities and signaling pathways, influencing cancer cell behavior and metabolism. Intracellular anatomical structure (GO:0005622), organelle (GO:0043226), cytoplasm (GO:0005737), and cell junction (GO:0030054) were among the common targets’ cellular component (CC) GO terms with gene ratios of 90.83%, 88.33%, 85%, and 25%, respectively (Table 8; Fig. 13B). These cellular components imply that AuNPs likely interact with multiple cellular compartments, affecting functions ranging from signaling to transport. The highest gene ratios of molecular function (MF) GO terms related to protein (GO:0005515; 95.83%), and small molecule (GO:0036094; 45%) binding (Table 8; Fig. 13C) underscore that AuNPs may influence protein interactions and enzyme activities critical for cancer progression.

**Table 8:** Top 15 GO terms of Cancer/AuNPs common targets.

	ID	Description	Count	GeneRatio	Ont*
1	GO:0050789	Regulation of biological process	110	110/120	BP
2	GO:0050794	Regulation of cellular process	107	107/120	BP
3	GO:0051716	Cellular response to stimulus	104	104/120	BP
4	GO:0044237	Cellular metabolic process	102	102/120	BP
5	GO:0071704	Organic substance metabolic process	99	99/120	BP
6	GO:0005622	Intracellular anatomical structure	109	109/120	CC
7	GO:0043226	Organelle	106	106/120	CC
8	GO:0005737	Cytoplasm	102	102/120	CC
9	GO:0016020	Membrane	80	80/120	CC
10	GO:0071944	Cell periphery	75	75/120	CC
11	GO:0005515	Protein binding	115	115/120	MF
12	GO:0036094	Small molecule binding	54	54/120	MF
13	GO:0097159	Organic cyclic compound binding	53	53/120	MF
14	GO:0044877	Protein-containing complex binding	39	39/120	MF
15	GO:0097367	Carbohydrate derivative binding	36	36/120	MF

* Ont: ontology; BP: biological process; CC: cellular component; MF: molecular functions.

**Table 9:** Top 15 KEGG pathways of Cancer/AuNPs common targets.

	ID	Description		Fold enrichment	z score	*P-*value	*P*.adjust	qvalue	Count
1	hsa05205	Proteoglycans in cancer		12.14	18.13	8.62E-26	2.05E-23	6.08E-24	31
2	hsa05418	Fluid shear stress and atherosclerosis		14.73	18.51	7.28E-24	8.66E-22	2.57E-22	26
3	hsa05215	Prostate cancer		17.93	18.98	3.05E-22	2.42E-20	7.16E-21	22
4	hsa05161	Hepatitis B		12.25	16.33	6.99E-21	4.16E-19	1.23E-19	25
5	hsa05210	Colorectal cancer		18.36	18.32	1.70E-20	6.75E-19	2.00E-19	20
6	hsa04933	AGE-RAGE signaling pathway in diabetic complications		16.60	17.76	1.67E-20	6.75E-19	2.00E-19	21
7	hsa04151	PI3K-Akt signaling pathway		7.28	13.75	2.90E-20	9.86E-19	2.92E-19	33
8	hsa05417	Lipid and atherosclerosis		9.61	14.44	5.76E-19	1.71E-17	5.08E-18	26
9	hsa05167	Kaposi sarcoma-associated herpesvirus infection		10.19	14.65	7.23E-19	1.91E-17	5.67E-18	25
10	hsa05235	PD-L1 expression and PD-1 checkpoint pathway in cancer		16.86	17.03	9.53E-19	2.27E-17	6.72E-18	19
11	hsa05163	Human cytomegalovirus infection		9.19	14.05	1.83E-18	3.96E-17	1.17E-17	26
12	hsa04066	HIF-1 signaling pathway		14.52	16.07	2.51E-18	4.98E-17	1.48E-17	20
13	hsa05213	Endometrial cancer		21.66	17.93	8.09E-18	1.48E-16	4.39E-17	16
14	hsa04210	Apoptosis		12.24	14.94	1.32E-17	2.24E-16	6.64E-17	21
15	hsa05212	Pancreatic cancer		17.63	16.51	3.26E-17	5.18E-16	1.53E-16	17

**Table 10:** KEGG modules of Cancer/AuNPs common targets.

	ID	Description	Fold Enrichment	z Score	*P*-value	*P*.adjust	qvalue
1	M00034	Methionine salvage pathway	14.72	3.61	0.07	0.12	0.12
2	M00035	Methionine degradation	14.72	3.61	0.07	0.12	0.12
3	M00002	Glycolysis, core module involving three-carbon compounds	13.58	3.45	0.07	0.12	0.12
4	M00165	Reductive pentose phosphate cycle (Calvin cycle)	11.77	3.17	0.08	0.12	0.12
5	M00003	Gluconeogenesis, oxaloacetate = > fructose-6P	9.81	2.85	0.10	0.12	0.12
6	M00001	Glycolysis (Embden-Meyerhof pathway), glucose = > pyruvate	6.79	2.26	0.14	0.14	0.14

**Fig. 14:**
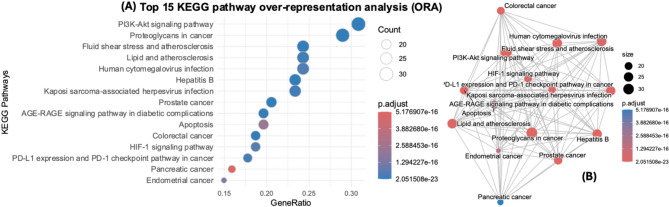
KEGG Pathways of Cancer/AuNPs’ target genes represented using dotplot (**A**) and mapplot (**B**).

**Fig. 15:**
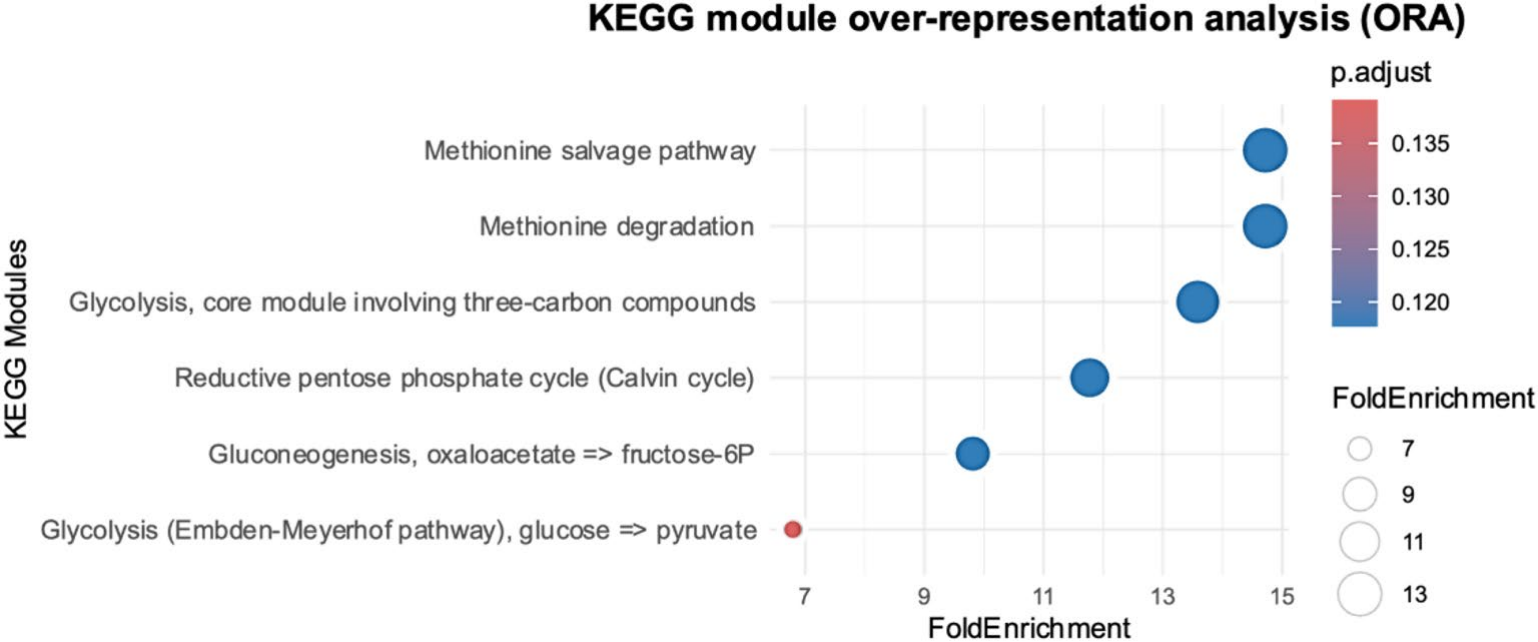
KEGG Modules of Cancer/AuNPs’ target genes.

**Fig. 16:**
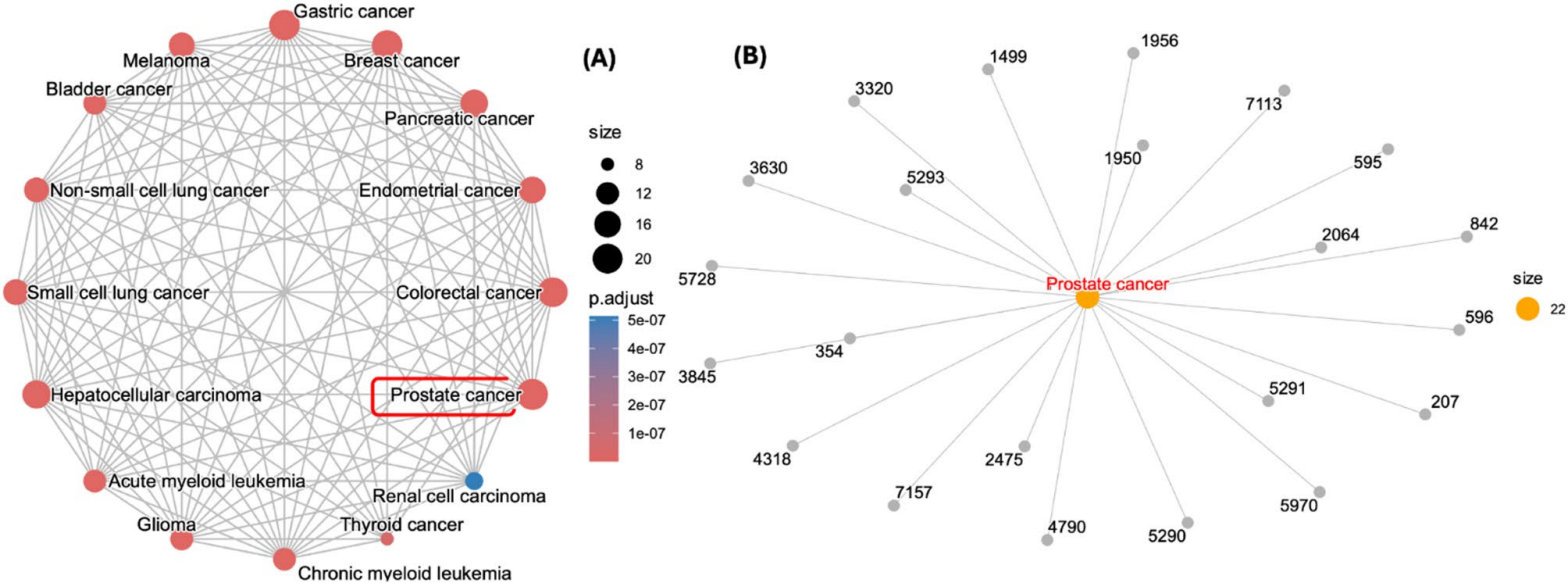
Mapping plot of the potential KEGG cancer types using a pairwise similarity matrix (**A**). Gene-concept network graph of the KEGG prostate cancer-related genes. Grey-colored nodes represent the 22 genes related to the KEGG prostate cancer pathway, expressed as ENTREZID (**B**).

KEGG pathway analysis of the 120 common targets revealed their potential involvement in different disease types and signaling pathways (Table 9; Fig. 14). Proteoglycans in cancer (hsa05205), fluid shear stress and atherosclerosis (hsa05418), prostate cancer (hsa05215), hepatitis B (hsa05161), colorectal cancer (hsa05210), AGE-RAGE signaling pathway in diabetic complications (hsa04933), and PI3K-Akt signaling pathway (hsa04151) were the most significant KEGG pathways at FDR *P*. adjust < 0.05, *P*-value < 0.05 and q-value < 0.2 (Fig. 15). Proteoglycans (hsa05205) in cancer and lipid/atherosclerosis (hsa05418) pathways are further related to tumor microenvironment and cancer cell invasiveness. Also, PD-L1 expression and PD-1 checkpoint pathway in cancer (hsa05235) reflects the AuNPs immunomodulatory potential critical for cancer immune evasion. While apoptosis (hsa04210) and HIF-1 signaling (hsa04066) pathways indicate that AuNPs can regulate programmed cell death and hypoxia responses, essential for tumor suppression. These pathways together suggest AuNPs target multiple oncogenic and survival mechanisms in PC cells. Among the resulted 16 human cancer types extracted with pathway ID of ‘hsa052XX’ (Fig. 16A), PC (hsa05215) showed the highly significant human cancer type, followed by colorectal cancer (hsa05210), highlighting the direct relevance of AuNPs’ impact on PC progression and treatment targets. Similarly, many recent studies have investigated the anticancer activity of gold-decorated nanoparticles against various cancer types, including head and neck squamous cell carcinoma (HNSCC)^129^, lung^130^, pancreatic^131^, prostatic^132,133^, breast^132,134–136^, and colorectal^137,138^ cancers.

**Fig. 17:**
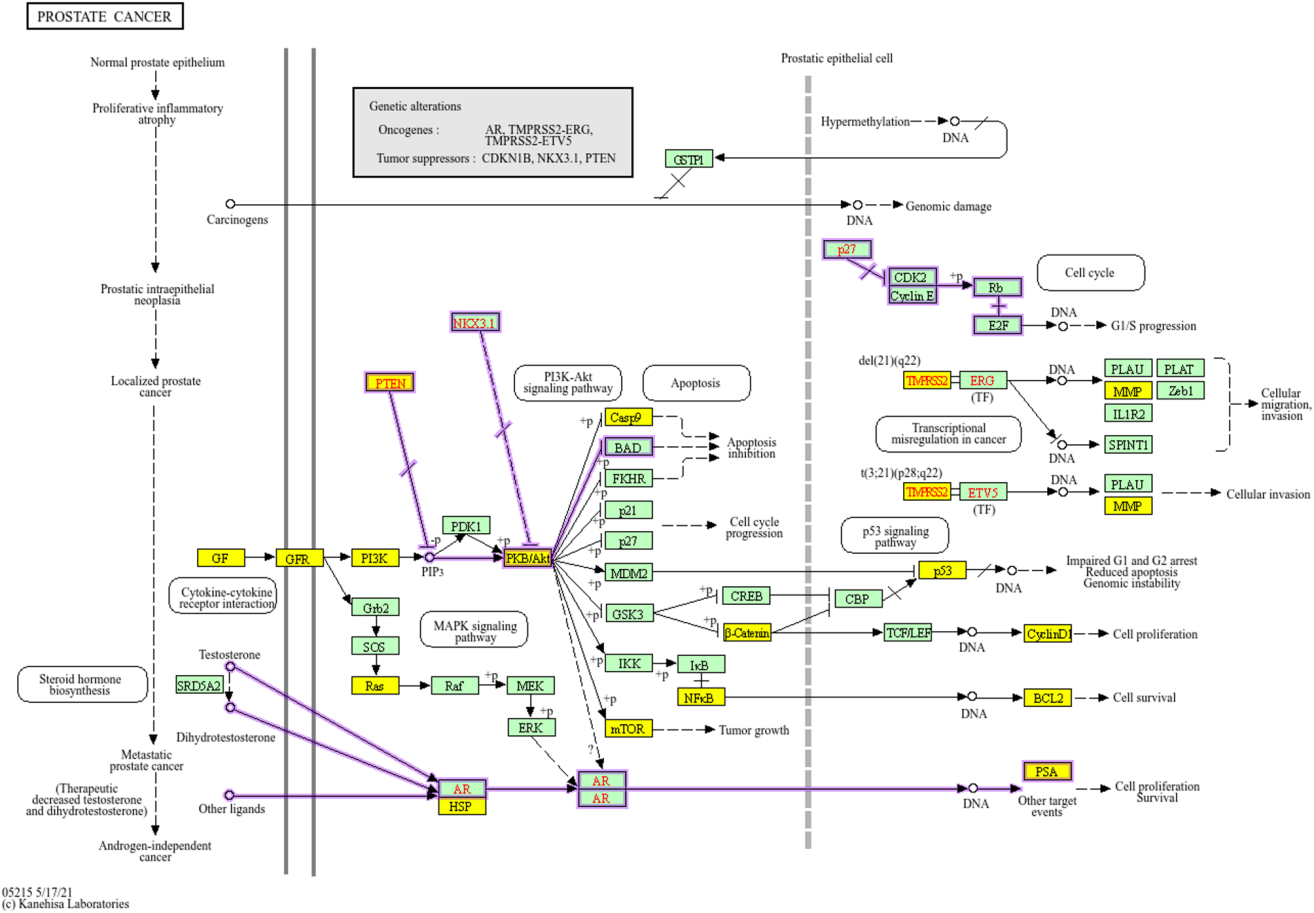
KEGG prostate cancer (hsa05215) pathway Map. Yellow highlights represent the potential core targets through which AuNPs would regulate prostate cancer incidence and propagation. Purple outlined arrows represent the three prostate cancer pathway modules, including cell cycle, PI3K signaling, and nuclear receptor signaling^147–149^. Permission to use these KEGG data was granted by the Kanehisa Laboratories under the CC BY 4.0 open-access license (Ref: 253169), which allows the use of this figure.

Our results showed that the most significant AuNPs’ anticancer therapeutic potential was for prostate cancer (hsa05215; Fold-Enrichment of 17.928; p. adjust of 2.42E-20) treatment through regulating 22 potential target genes (Fig. 16B), including AKT1, BCL2, CASP9, CCND1, CTNNB1, EGF, EGFR, ERBB2, HSP90AA1, INS, KLK3, KRAS, MMP9, MTOR, NFKB1, PIK3CA, PIK3CB, PIK3CD, PTEN, RELA, TMPRSS2, and TP53. Among these 22 genes, 6 carcinogenic hubs were previously identified as highly PPI-connected nodes with a degree centrality ≥ 50, including AKT1, BCL2, CTNNB1, EGFR, MMP9, and TP53. Moreover, the KEGG pathway map (Fig. 17) revealed the involvement of these 22 core targets in prostate pathogenesis through oncogenic pathway modules, including apoptotic evasion, cell cycle dysregulation, nuclear receptor, and PI3K/Akt signaling. The direct targeting of PC (hsa05215) and PI3K/AKT signaling (hsa04151) pathways suggests interference with oncogenic signaling critical for PC progression^139^. The inhibition of PI3K/Akt prevents downstream pro-survival signals, leading to cell cycle arrest and apoptosis, which is supported by the enrichment of apoptosis pathways (hsa04210). Thus, aberrant PI3K/AKT signaling drives therapeutic resistance in prostatic carcinogenesis and propagation by promoting cell survival and proliferation^140^. AuNPs can suppress autophagy by targeting the PI3K/AKT/mTOR pathway^141^. They can also regulate the tumor suppressor PTEN gene expression (see Fig. 17), which acts as a direct antagonist to the oncogenic PI3K signaling^142^. However, the reciprocal interaction between PI3K/Akt and androgen receptor (AR) pathways, where the inhibition of PI3K/Akt/mTOR signaling may result in AR transcriptional upregulation through activating membrane signaling proteins like HER3^140^, would provide an alternative way for prostatic carcinogenesis. Thus, AuNPs could provide a co-targeting strategy involving both PI3K/Akt/mTOR and AR pathways to improve the treatment outcomes in aggressive prostate cancer. Additionally, modulating the expression of PD-L1/PD-1 checkpoint pathway (hsa05235) suggests that AuNPs may enhance anti-tumor immunity by migrating cancer immune evasion^143,144^.

Furthermore, AuNPs affect six potential KEGG metabolic modules of the tested targets (Table 10; Fig. 15) included methionine salvage pathway (M00034), methionine degradation (M00035), glycolysis core module, reductive pentose phosphate cycle (Calvin cycle; M00165), gluconeogenesis (M00003), and Embden-Meyerhof glycolysis pathway (M00001), reflecting an impact on tumor cell metabolism. Perturbation of these pathways can disrupt energy production and biosynthetic precursors, thus inhibiting tumor growth. Similarly, it has been reported that AuNPs disrupt PC metabolism and anabolic processes via modulating glycolysis^145^ and methionine^146^ metabolism. This novel insight into targeting metabolic reprogramming provides an innovative therapeutic strategy, differentiating from existing PC treatments focused mainly on hormonal and receptor modulation.

The current *in silico* computational analysis, besides *in vitro* investigation, highlights the critical role of bioinformatics in guiding experimental cancer nanomedicine research, exploring the Au-NPs’ anti-prostatic mechanistic role and their potential implications for future therapeutic strategies through multi-faceted molecular modulation of oncogenic, metabolic, and immune pathways. Their binding to key protein targets involved in survival and proliferative pathways, inhibiting the PI3K/Akt pathway, inducing apoptosis, metabolic reprogramming, tumor microenvironment, and immune checkpoint modulation are collectively inhibiting PC growth and progression. Additionally, AuNPs’ biocompatibility, selectivity, minimalized side effects, and ability to be functionalized make them suitable for targeted cancer therapy. Au-NPs have also been demonstrated to selectively target cancer cells due to their size and shape, which facilitate accumulation in tumor tissues. The selective site-specific targeting and cellular uptake of AuNPs is crucial for their efficacy^150–152^, minimizing damage to healthy cells. Moreover, Functionalized Au-NPs conjugates with molecules such as antibodies and drugs (e.h. DOX) enhance their specificity and effectiveness, allowing for precise targeting of cancer cells and direct delivery of therapeutic agents^153^.

## Conclusion

The present study successfully demonstrated a rapid, eco-friendly biosynthesis of AuNPs using the red alga *Jania rubens*, yielding well-characterized, stable Jan-AuNPs with robust biomedical potential. Phytochemical characterization of the algal extract revealed a diverse and abundant biochemical rich composition, including saturated fatty acids (particularly palmitic and stearic acids), a notably high total phenolic content (299.82 mg GAE/g DW), flavonoids (35.61 mg QE/g DW), and soluble carbohydrates (166 mg GE/g DW). This abundant phytochemical composition is proposed to serve as a source of natural reducing, capping, and stabilizing agents, thereby driving the green synthesis process and conferring physicochemical integrity to the resulting Jan-AuNPs. Comprehensive physicochemical characterization confirmed the formation of predominantly spherical, crystalline nanoparticles with an average diameter of 16.26 nm, a surface plasmon resonance peak at 549 nm, and a zeta potential of − 28 mV, indicative of favorable colloidal stability. FTIR analysis underscored the critical role of algal-derived biomolecules in mediating nanoparticles formation and capping, while FCCCD-guided optimization elevated the biosynthesis yield to 289 µg/mL under defined conditions of pH 7, 60 °C, 3 h incubation, and 300 µg/mL gold ion concentration. A synergistic *in silico*, *in vitro*, and *in vivo* investigative pipeline provided compelling evidence for the anticancer efficacy of Jan-AuNPs. Jan-AuNPs exerted potent and selective cytotoxicity against PC3 prostate cancer cells while preserving the viability of normal HFB4 cells, reflecting their favorable safety profile. Furthermore, the combination of Jan-AuNPs with doxorubicin achieved a remarkable tumor growth inhibition rate of 97.33% in the *in vivo* EAC mouse model. *In silico* computational analysis explores the Jan-AuNPs’ anti-prostatic mechanistic role by targeting crucial biological processes, oncogenic signaling and metabolic pathways. GO/KEGG pathway enrichment analysis provided meaningful mechanistic insight into their multi-targeted mode of action of Jan-AuNPs against aggressive prostate cancer. These findings establish *Jania rubens*-mediated biosynthesis as a sustainable and scalable green platform for the production of bioactive AuNPs with well-defined physicochemical properties. Jan-AuNPs emerge as a safe, efficacious, and multi-targeted nanotherapeutic candidate warranting further translational investigation toward clinical application in the treatment of prostate cancer.

### Limitations

Despite the promising results, certain limitations should be acknowledged. The *in silico* predicted molecular targets and signaling pathways require additional experimental validation, and the *in vivo* assessment was restricted to the EAC model, which does not fully reflect the clinical characteristics of human prostate cancer. Evaluation in prostate cancer-specific animal models, such as xenograft or orthotopic models using PC3 cells, would provide more clinically relevant evidence. Additionally, long-term safety and biodistribution, and pharmacokinetic profiles of Jan-AuNPs were not evaluated. These limitations outline clear directions for future research for the continued development of Jan-AuNPs as a clinically viable nanotherapeutic candidate.

## Data Availability

All data generated or analyzed during this study are included in this article.
